# Dynamic processes of mindfulness-based alterations in pain perception

**DOI:** 10.3389/fnins.2023.1253559

**Published:** 2023-11-09

**Authors:** Chen Lu, Vera Moliadze, Frauke Nees

**Affiliations:** Institute of Medical Psychology and Medical Sociology, University Medical Center Schleswig-Holstein, Kiel University, Kiel, Germany

**Keywords:** mindfulness, analgesia, chronic pain, pain perception, brain signature

## Abstract

Mindfulness-based processes have been shown to enhance attention and related behavioral responses, including analgesia, which is discussed as an effective method in the context of pain interventions. In the present review, we introduce the construct of mindfulness, delineating the concepts, factors, and processes that are summarized under this term and might serve as relevant components of the underlying mechanistic pathways in the field of pain. We also discuss how differences in factors such as definitions of mindfulness, study design, and strategies in mindfulness-based attention direction may need to be considered when putting the findings from previous studies into a whole framework. In doing so, we capitalize on a potential dynamic process model of mindfulness-based analgesia. In this respect, the so-called mindfulness-based analgesia may initially result from improved cognitive regulation strategies, while at later stages of effects may be driven by a reduction of interference between both cognitive and affective factors. With increasing mindfulness practice, pathways and mechanisms of mindfulness analgesia may change dynamically, which could result from adaptive coping. This is underlined by the fact that the neural mechanism of mindfulness analgesia is manifested as increased activation in the ACC and aINS at the beginner level while increased activation in the pINS and reduced activation in the lPFC at the expert level.

## 1. Introduction

### 1.1. Pain and pain perception

Pain is defined as “an unpleasant sensory and emotional experience associated with actual or potential tissue damage, or described in terms of such damage” (Merskey and Bogduk, 1994). Pain perception is, therefore, a complex process that is comprised of various components including not only the sensory system, but also cognitive, and affective-motivational dimensions (Apkarian et al., 2011). A variety of cognitive processes have been shown to influence pain perception and bias nociceptive processing, both on the behavioral (Perlman et al., 2010; Wetherell et al., 2011; Ehde et al., 2014; Diotaiuti et al., 2021; Vencatachellum et al., 2021) as well as the neural level in the human brain (Rainville et al., 1997; Wager et al., 2004; Vanhaudenhuyse et al., 2009; Gard et al., 2012; Jensen et al., 2012; Zeidan et al., 2015; Mioduszewski et al., 2020). One of those processes is clearly attention.

Studies have demonstrated that when attention is diverted from pain, pain intensity may decrease (Bantick et al., 2002; Terkelsen et al., 2004; Chan et al., 2012), accompanied by increased activation in the anterior cingulate cortex (ACC) (Bantick et al., 2002). This may reflect a top-down modulation of pain-related areas and is thought to be initiated by increased activation in the prefrontal cortex (PFC) (Petrovic et al., 2000; Wager et al., 2004; Wiech et al., 2006).

In line with our daily experience, focusing on pain may lead to an increase in pain intensity (Quevedo, 2007). While such findings apply to healthy study populations, it may be additional altered in chronic pain patients (Hadjistavropoulos et al., 2000): chronic pain patients who were particularly health-anxious reported less anxiety and pain when they focused on the physical sensations. Moreover, a different pattern may emerge in individuals, who are trained in focusing their attention to specific and related attention shifts. This is then also of interest in the context mindfulness-based strategies, where strong individual differences may exist in terms of performance duration and frequency. Indeed, researchers found that mindfulness experts reported lower pain when keeping their attention on the site of pain compared to novices with no experience with mindfulness (Gard et al., 2012).

### 1.2. Mindfulness

Mindfulness has its roots in the Buddhist tradition of meditation and is described as: “Consciousness that arises through purposeful attention, in the present moment, without judgment, to the development of experience from moment to moment” (Baer, 2003; Kabat-Zinn, 2003). It is mainly defined as a mental state achieved by purposeful awareness of the present moment with an accepting and nonjudgmental attitude (Brown and Ryan, 2003; Kabat-Zinn, 2003; Germer & Neff, 2013). While this reflects the ambition to provide a clear and precise description, it also becomes clear that mindfulness is a rather broad term integrating components of the state of mindfulness (a changeable state of being), the process of achieving mindfulness and the trait mindfulness (a stable quality with a fairly normal distribution across the population) (Kabat-Zinn, 2003; Cahn and Polich, 2006; Shapiro & Carlson, 2009; Lu et al., 2014; Scavone et al., 2020). This means mindfulness can be understood as both a process and the endpoints of this process. The plurality of interpretations of mindfulness as a concept may also be a non-negligible reason for the inconsistency in the findings of previous studies on mindfulness analgesia. Therefore, for ease of understanding, the word “mindfulness” shown in this review mainly takes the position of viewing mindfulness as a state of being, if not otherwise specified.

### 1.3. Clinical effects of mindfulness in chronic pain

Regarding the clinical efficacy of mindfulness in pain management, a systematic review and meta-analysis of mindfulness-based training strategies for chronic pain effectiveness revealed that mindfulness was associated with a modest reduction in pain when compared to all types of control groups (Hilton et al., 2017). Similarly, another systematic review and meta-analysis investigating the effectiveness of mindfulness for adult low back pain (LBP) found a significant positive impact on post-treatment pain intensity, but this effect was no longer significant during follow-up (Schmidt and Pilat, 2023). These results of the presented clinical studies can be seen as first evidence on the analgesic effect of mindfulness (Braden et al., 2016; Mioduszewski et al., 2020; Day et al., 2021; Smith et al., 2021; Parisi et al., 2022). What is still lacking, however, is information and data on the duration of the achieved effects and also the quality of some studies have been raised due to issues such as small sample size, inconsistent conceptualizations of mindfulness, and lacking of active control groups [for more randomized controlled trials (RCT) studies showing the clinical effects of mindfulness on chronic pain, see Supplementary Table S1]. Researchers have therefore emphasized the need for more meticulously designed, rigorous, and large-scale RCTs in the future to more precisely and adequately estimate the efficacy of mindfulness in chronic pain (Hilton et al., 2017; Wielgosz et al., 2019; Schmidt and Pilat, 2023).

### 1.4. Effects on brain structure and function induced by mindfulness-based processes

**Figure 1:**
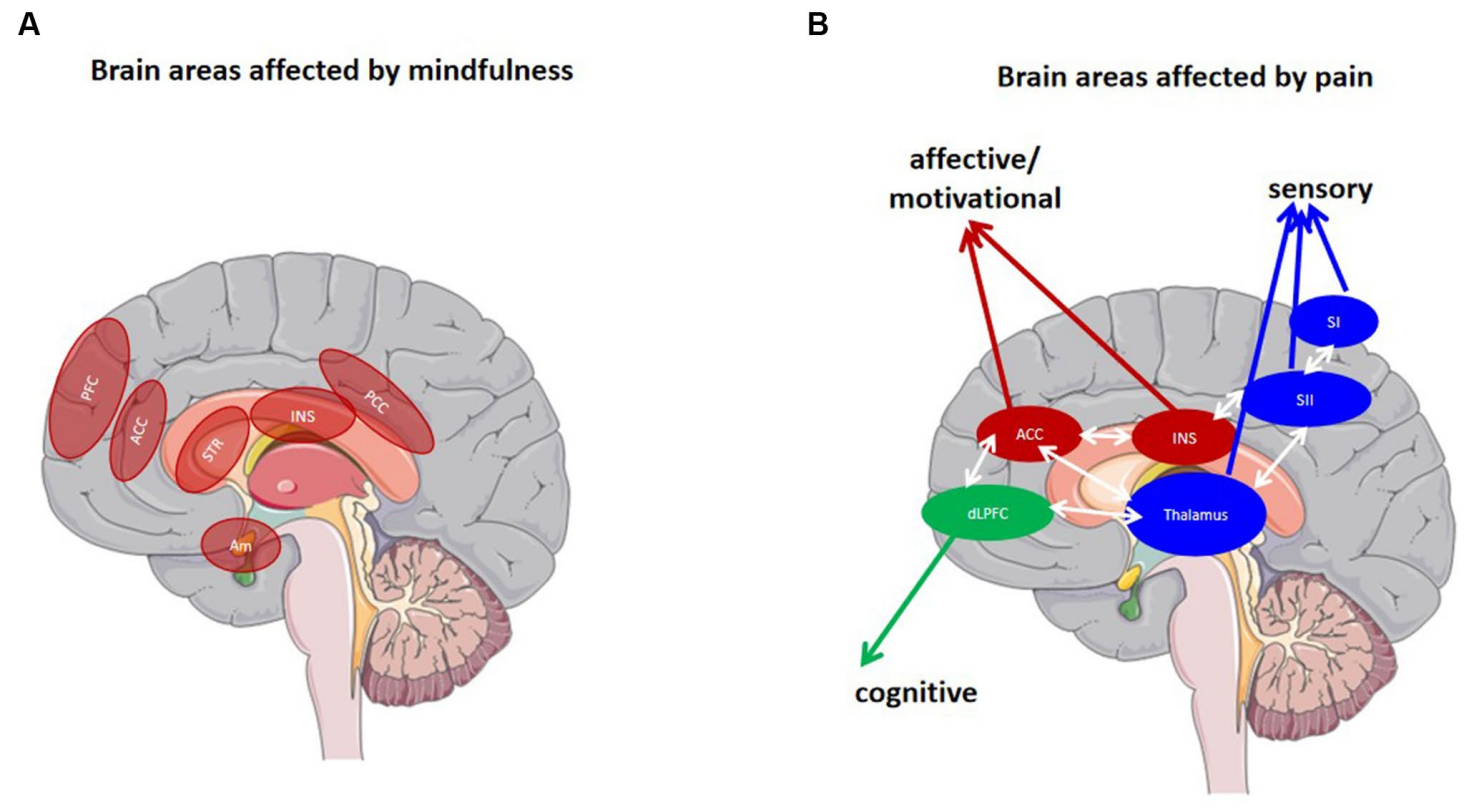
Brain areas involved in pain (processing) and mindfulness. **(A)** Regions involved in the mindfulness mediation [Adapted Tang et al. (2015)]. **(B)** Regions affected by pain. Blue areas represent common structures in the sensory/discriminative (lateral) pain pathway (thalamus, SI, SII). Red areas represent common structures identified in the affective/motivational pain pathway (ACC, INS). The green region represents one component of the cognitive/evaluative pain system (dlPFC). The white arrows represent multiple cortical connections between regions [Adapted by Monroe et al. (2015)]. PFC prefrontal cortex, ACC anterior cingulate cortex, INS Insula, PCC posterior cingulate cortex, Am amygdala, STR Striatum. Parts of the figure were drawn by using pictures from Servier Medical Art. Servier Medical Art by Servier is licensed under a Creative Commons Attribution 3.0 Unported License (https://creativecommons.org/licenses/by/3.0/).

Some studies (Hölzel et al., 2011; Jensen et al., 2014) assume that mindfulness can cause lasting, trait-related changes in the brain. Individuals, who experienced long-term mindfulness training, showed increased grey matter and cortical thickness in the hippocampus, brainstem, anterior and posterior insula, middle cingulate cortex (MCC), parietal cortex, and PFC when compared to novices (see Figure 1A). Some of these brain regions are also involved in pain processing (see Figure 1B; Pernet et al., 2021). It has been demonstrated that the thickness of certain brain regions is positively associated with the duration or amount of mindfulness training, and negatively correlated with sensitivity to pain stimuli induced experimentally (Grant et al., 2010). This may apply particularly to the MCC, which participates in pain inhibition (Rainville et al.,1999), and a reduction in grey matter volume in the MCC has commonly been observed in patients with chronic pain (May, 2008). Therefore, it can be hypothesized that these brain structures may constitute a correlate of mindfulness training. This provides information when it comes to brain-based pain treatments to intervene and prevent the development of chronic pain, particularly in patient subgroups exhibiting corresponding maladaptive brain-behavior changes. Remarkably, a study also reported on structural changes in the brain, such as increased grey matter in the hippocampus and parietal regions, following a brief mindfulness training, not only in individuals who are experts in mindfulness (Hölzel et al., 2011). This suggests that mindfulness training may have potential benefits for novice practitioners and could thus serve as a strategy for patients managing their chronic, and potentially chronic pain.

Moreover, it is also important to note that mindfulness did not only result in structural changes in brain areas, but also seems to trigger changes in the connections between neural networks (Su et al., 2016). A recent meta-analysis systematically examined the effects of mindfulness on resting state functional connectivity (rsFC), as a neural marker of cognitive and emotion regulation (Rahrig et al., 2022). Significantly greater rsFC between the left middle cingulate, located within the salience network (SN), and the posterior cingulate cortex (PCC), a focal hub of the default mode network (DMN), was found for mindfulness treatment compared to a control treatment. Moreover, increased connectivity between the PCC (as part of the DMN) and dorsolateral prefrontal cortex (dlPFC; as part of the frontoparietal network; FPN), as well as increases in connectivity between the dorsolateral anterior cingulate cortex (ACC; as part of the SN) and anterior insula (aINS; as part of the SN), have been reported (Sezer et al., 2022). These networks are involved in the flexible control of internally-oriented attention processes, which may thus be facilitated by mindfulness.

This is also in line with brain imaging in long-term persistent pain, where dysregulations of cortical brain circuits involving regions such as the PFC, including the ACC, insula, amygdala, and nucleus accumbens (Yang and Chang, 2019), have been reported. Participants with chronic pain were shown to exhibit increased functional connectivity between the aINS and dACC after mindfulness training compared to healthy individuals (Su et al., 2016). The dACC regions are related to attention (Bush, 2011), which is a major component of mindfulness (Lutz et al., 2008) and has been shown to modify pain processing (Kabat-Zinn, 1982). This indicate that mindfulness may be used to trigger pain relief processes by improving attention flexibility, potentially mainly driven by the dACC (as part of the SN) and aINS (as part of the SN) connectivity.

### 1.5. Possible brain-behavior pathways of mindfulness-based analgesia and inconsistencies in the previous studies

Although researchers (Dimidjian and Linehan, 2003; Shapiro et al., 2006) have not yet reached a consensus on the definition of mindfulness, one thing for sure is that “ability to return to the present moment” (attention) and the “curious, open, and receptive attitude” (mindful attitude) are the two core components of mindfulness (Duan, 2014). The analgesic effect of mindfulness can be achieved by changing pain anticipation (Brown and Jones, 2010; Gard et al., 2012; Vencatachellum et al., 2021; Sezer et al., 2022), pain catastrophizing (Jensen et al., 2014; Garland and Howard, 2018; Vencatachellum et al., 2021), pain unpleasantness (Brown and Jones, 2010; Perlman et al., 2010; Zeidan et al., 2011, 2015; Gard et al., 2012; Lutz et al., 2013), and pain intensity (Grant and Rainville, 2009; Zeidan et al., 2011, 2015). Mindfulness might exert its impact by decreasing the interference of experience-based top-down cognitive and affective processing with ongoing pain processing, which could lead to a reduction in pain intensity. In addition, maybe due to the inherent attention-captivating nature of pain, paradigms that involve increasing cognitive load through inhibition of “habitual” processes, such as the Stroop task, have been suggested to potentially be ineffective in diverting attention away from pain (Wiech et al., 2008). In contrast to other methods of directing attention, it can be hypothesized that mindfulness-based processes that involve curiosity, openness, and acceptance are a more effective approach to directing focus away from pain and instead toward a specific task (e.g., focusing on breathing). Indeed, mindfulness has been shown to result in even stronger effects than single distraction or other attention-related mechanisms (e.g., placebo) (Zeidan et al., 2015).

It is important to note that when looking again at the neural mechanisms with respect to different attention modulations of pain and mindfulness-based attention regulation, the contradictions in the findings of previous studies can be clearly seen. Some researchers have proposed that mindfulness has similar pathways and neural mechanisms in comparison to other attention-related pain interventions (Zeidan et al., 2012) and that the effect is based on similar cognitive regulatory components. However, others rather suggest that mindfulness involves other and/or additional brain areas (Zeidan et al., 2011, 2015) and that this may be based on an increased sensory processing of the pain and in a replacement of typical attempts to exert more cognitive control over the pain (Gard et al., 2012). This may appear together with a distinct state of cognitive disengagement and nonjudgmental sensory awareness (Zeidan et al., 2012) and may be related to decreased activation in the lateral prefrontal cortex (lPFC) and increased activation in the right pINS during stimulation (Gard et al., 2012). This seems to imply that a new model to explain this inconsistency of findings is essential.

This review aims to analyze and compare previous research findings to understand the potential reasons for the inconsistency in study conclusions. Based on this analysis, a theoretical model is proposed to partially elucidate this inconsistency. We used the search terms “mindfulness,” “pain” in the databases of PubMed, and Google Scholar. For clinical studies of mindfulness interventions in chronic pain patients the inclusion criteria were as follows: studies were required to involve mindfulness meditation, either as an adjunctive or monotherapy; studies testing other meditation interventions such as yoga, tai chi, qigong, and transcendental meditation techniques without reference to mindfulness were excluded. For studies of experimentally induced pain our inclusion criteria were as follows: subjects were required to engage in mindfulness meditation during the experiment or the experimental group of subjects should have been mindfulness experts. It should be noted that our review is an opinion-based review rather than a systematic review or a meta-analysis study. Therefore, the selection and organization of references are based on the presentation of viewpoints and models in this review.

## 2. The impact of mindful attitude and attention on pain perception

Mindful attitude and attention are two assumed core components of mindfulness, that may also be the main components when it comes to mindfulness-based analgesia (Duan, 2014).

### 2.1. Effects of mindful attitude on pain perception

One important aspect of the analgesic effects of mindfulness may involve the attainment of a constant state of equanimity toward painful sensations and/or negative thoughts by maintaining a mindful attitude that involves being aware of these experiences without engaging in reactive responses or attempts to alter them (Hayes et al., 1999). Wang et al. (2015) showed that, when individuals re-experience painful stimuli, the control group used more spontaneous distraction to avoid these painful stimuli, whereas the mindfulness training group shifted their attitudes toward pain and thus achieved a better acceptance of these stimuli (Wang et al., 2015). This process of accepting the pain might then also affect processes critical for pain chronification, like for example fear learning processes, and could in turn reduce hyperalgesia responses (Taylor et al., 2018). An “accepting, non-avoidant” mindful attitude can thus change responses during the exposure to pain, and assist common behavioral therapeutic strategies like facilitating pain-related fear extinction learning (Martinez-Calderon et al., 2019; Diotaiuti et al., 2023). This may lead to a larger effect size and stronger pain relief.

### 2.2. Effects of mindful attention on pain perception

Attention is still regarded as the essential process and critical mechanistic element for the effectiveness of mindfulness (Shapiro et al., 2006; Maser, 2012), even though the fact that an accepting mindful attitude has also been demonstrated to be a significant outcome of mindfulness training (Brown and Ryan, 2003; Baer et al., 2004) and is effective in enhancing pain tolerance (McCracken et al., 2005; Masedo and Rosa, 2007; Kohl et al., 2012) and significantly reducing pain intensity and pain unpleasantness (Masedo and Rosa, 2007). Jha et al. (2007) explored the effects of mindfulness on specific attention functions (orientation, vigilance, and conflict monitoring) and showed that mindfulness can improve attention-related behavior responses by improving the functioning of specific attention subsystems (Jha et al., 2007). Maser (2012) further demonstrated that the positive effects of mindfulness training observed in patients with chronic pain may be due to less orienting of attention toward pain sensations and indicated that mindfulness training increased individuals’ attention flexibility (Maser, 2012). The other studies, although not directly focused on the effects of mindfulness training on attention, have indirectly demonstrated that attention is critical to the mechanism of mindfulness (Watkins et al., 2000; Carmody and Baer, 2008; Grant and Rainville, 2009; Erisman and Roemer, 2010; Perlman et al., 2010). These studies suggest that mindfulness can increase individuals’ resistance to internal and external negative stimuli and experiences. Some researchers (Williams, 2010; Peng and Ju, 2013) believe that this resistance is achieved through a shift in individuals’ state of mind through mindfulness, whereby otherwise negative experiences associated with the ego are perceived as objective and do not bring about excessive subjective evaluations and emotions. This shift in individuals’ state of mind could also be related to the increased attention flexibility caused by mindfulness training, which enables individuals to focus on the task they want to concentrate on, rather than being uncontrollably drawn to the pain sensation.

Overall, the increased attention flexibility induced by mindfulness training allows individuals to maintain awareness of the present state/moment including a pure experience of their feelings, and to develop the attitude of openness and acceptance based on this. And the mindful attitude in turn helps attention to be better maintained on what is being experienced at the moment, whether the experience is positive or not.

## 3. Into a dynamic model of mindfulness-based analgesia

Some researchers pointed out that the early stages of mindfulness practice may improve the ability to orient attention by selecting specific information from multiple sensory stimuli and attending to conflicts between different regional activities (Tang et al., 2015). In the field of mindfulness analgesia, this can be interpreted as indicating that in the early stages of mindfulness practice, the individual achieves pain relief by consciously focusing attention on the ongoing task (e.g., focusing on breathing). Mindfulness practice allows them to better reduce spontaneous orienting responses to painful stimuli, which may achieve by increased cognitive regulation. With the increased practice of mindfulness, the brain may redirect neural activity and effort from default-mode regions to cooperating attention and frontoparietal regions as a nonlinear function of the amount of practice (Brefczynski-Lewis et al., 2007; Lutz et al., 2008; Manna et al., 2010; Tang et al., 2015; Falcone and Jerram, 2018). Redirecting cognitive resources to attention and executive function is consistent with de-automatizing emotional and cognitive reactions that have become habitual and spontaneous (Fiske, 1989; Amanda et al., 2014). Increased practice resulted in more-efficient transitions to mindful attention, suggesting the possibility for a more reflexive and less effortfully controlled mindset (Denny et al., 2015). That is, as the mindfulness level improve with the increased amount of practice, the achievement of mindfulness analgesia will no longer require dependent cognitive regulation but will be achieved in a way that spontaneously dissociates sensory processing from cognitive-affective processing. The moderating role for the mindfulness level between mindfulness training and related effects have also been found by other researchers (Xu et al., 2015).

**Figure 2:**
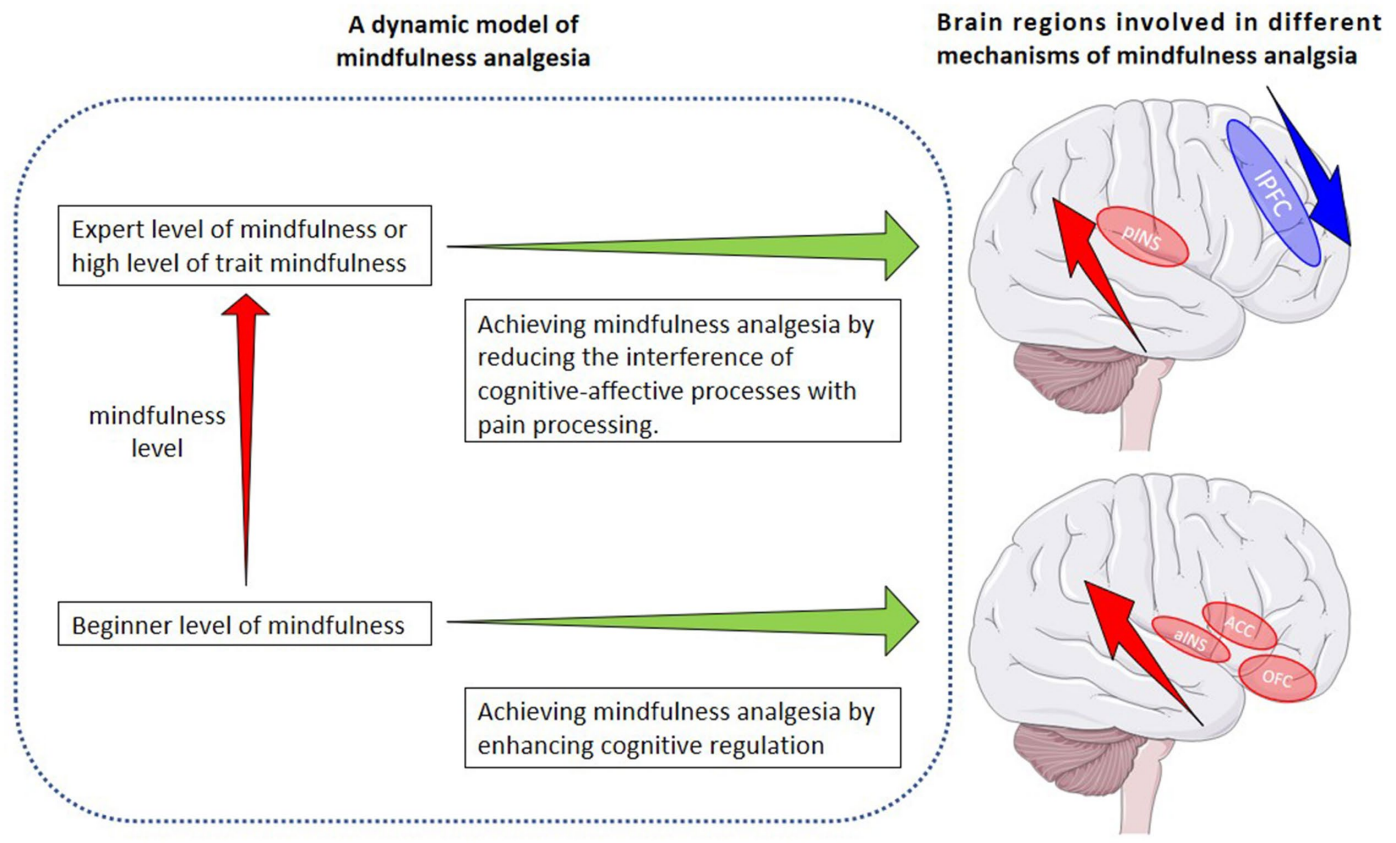
A dynamic model of mindfulness analgesia and brain regions involved in two different mechanisms of mindfulness analgesia. ACC anterior cingulate cortex, aINS anterior insula, OFC orbitofrontal cortex, pINS parietal insula, lPFC lateral prefrontal cortex. Parts of the figure were drawn by using pictures from Servier Medical Art. Servier Medical Art by Servier is licensed under a Creative Commons Attribution 3.0 Unported License (https://creativecommons.org/licenses/by/3.0/).

These may allow us to present a *dynamic model*: initially, mindfulness analgesia is achieved through enhanced cognitive regulation. As the mindfulness level increases, the analgesic effect of mindfulness will no longer rely on cognitive control, but may rather be achieved by dissociating sensory processing with cognitive-affective processing (see Figure 2). At this point, pain processing tends to be based solely on the sensory processing of the current stimulus, with little interference from cognitive-affective factors.

The dynamic model of mindfulness-based analgesia suggested by us appears to receive some indirect support from previous studies (Fiske, 1989; Brefczynski-Lewis et al., 2007; Lutz et al., 2008; Manna et al., 2010; Zeidan et al., 2012; Amanda et al., 2014; Denny et al., 2015; Tang et al., 2015; Zeidan and Vago, 2016; Falcone and Jerram, 2018; Sezer et al., 2022; Zhou et al., 2023). For example, a recent study (Sezer et al., 2022), in which the analgesic mechanisms of trait mindfulness and mindfulness training have been investigated, found that mindfulness training triggered changes in subjects’ functional brain connectivity different from those triggered by trait mindfulness. Although both manipulations of mindfulness resulted in the decoupling of the SN from the DMN, trait mindfulness was associated with the decoupling of the FPN and DMN, whereas mindfulness training was associated with enhanced functional connectivity in the FPN and DMN. This may indicate that, after a period of mindfulness training, the individual’s mindfulness level has improved but has not yet reached the level of an expert or an individual with a high level of trait mindfulness. In this case, the reduction in reported pain intensity/unpleasantness by the individual may rely on different mechanisms compared to those reported by experts (Gard et al., 2012) or subjects with a high level of trait mindfulness (Zeidan et al., 2018). The reduction in scores of pain unpleasantness triggered by a short period of mindfulness training is associated with increased activation of cognitive-related brain areas, indicating increased cognitive modulation during pain processing. As mindfulness training progresses and an individual’s mindfulness level further increases (Zeidan et al., 2011, 2015), eventually reaching a qualitative change: pain processing solely based on the current pain without cognitive-affective process interference starts to dominate, indicating the moderating role of mindfulness level in pain processing.

This theoretical model also partly explains the inconsistency between the conclusions of previous studies. There are two common research design in previous studies on the mechanisms of mindfulness analgesia: (1) comparing mindfulness experts (or subjects with a high level of trait mindfulness) with novices (or subjects with a low level of trait mindfulness); (2) comparing mindfulness training groups with control groups (Zeidan et al., 2011, 2015, 2018; Gard et al., 2012; Lutz et al., 2013). The latter usually result in significantly lower pain intensity scores for subjects in the mindfulness group compared to the control group (Zeidan et al., 2011, 2015), while the former usually not (Gard et al., 2012; Lutz et al., 2013). Furthermore, even they both reported reduced pain unpleasantness scores in the mindfulness group, the neural mechanisms behind the reduced scores were the complete opposite. The latter is thought to be related to the increased activation in brain regions associated with cognitive regulation (Zeidan et al., 2011, 2015), whereas the former is thought to be related to reduced activation (or deactivation) of brain regions associated with cognitive control (Gard et al., 2012; Zeidan et al., 2018). The differences in these findings could also be explained by the difference in the mindfulness level between mindfulness experts and the beginners.

## 4. Limitations and complement to the dynamic model

Although the dynamic model of mindfulness-based analgesia can partially account for the inconsistencies found in previous research, it is possible that additional factors also contribute to the discrepancies observed across studies.

### 4.1. Mindfulness-based training strategies might not be sensitive to target neural mechanisms

The implementation of mindfulness-based training strategies as manipulations of mindfulness in experiments could also potentially contribute to the mixed findings observed in previous studies. It allows to introduce additional non-mindful processes or to focus on a specific mindfulness-related factor, while controlling other potentially confounding factors. Specifically, in research on mindfulness-based pain interventions, training procedures that include the concept of mindfulness are often combined with other intervention strategies (e.g., yoga, education, and evidence-gathering). These strategies also partly contain aspects of mindfulness as a theoretical concept and thus did not allow to draw clear mechanistic conclusions. Moreover, some previous studies have focused mainly on one component of mindfulness (mindful attitude or attention) instead of comparing different components to come to a more fine-grained conclusion. Finally, many psychological processes may come into play co-determining, for example, facilitating, mindfulness-based training effects (e.g., reappraising the pain sensations as non-dangerous, empathy and compassion) (Singer and Klimecki, 2014; Berry et al., 2020; Ashar et al., 2022). For instance, mindfulness-based stress reduction (MBSR) (Kabat-Zinn, 1990), mindfulness-based cognitive therapy (MBCT) (Segal et al., 2002) and Mindfulness-Oriented Recovery Enhancement (MORE) (Garland and Howard, 2018; Parisi et al., 2022) and Pain Reprocessing Therapy (PRT) (Ashar et al., 2022) are mindfulness-based training strategies which have been widely used in clinical intervention. Numerous studies have demonstrated their impact on pain perception and efficacy in pain relief (Braden et al., 2016; Mioduszewski et al., 2020; Day et al., 2021; Santarnecchi et al., 2021; Smith et al., 2021; Ashar et al., 2022; Crisp et al., 2023) (for RCT studies about mindfulness interventions in acute and chronic pain see Supplementary material). All these procedures emphasize formal and informal mindfulness practices, while also incorporating additional processes beyond mindfulness. It may be for this reason that some researchers (Perlman et al., 2010) have argued that studies based on these mindfulness-based training strategies are difficult to interpret and lack a precise identification of the underlying mechanisms of mindfulness analgesia. Therefore, future research should consider employing manipulations more carefully in terms of potential con-founders and should make clear whether mindfulness is used as an intervention strategy or an experimental manipulation to minimize the interference of non-targeted, but mindful-related processes.

In addition, some researchers investigated how mindfulness-based training strategies are affected by specific factors such as reappraising the causes and threat value of pain, focusing attention on pleasant events, and self-compassion (Singer and Klimecki, 2014; Garland and Howard, 2018; Berry et al., 2020; Ashar et al., 2022; Parisi et al., 2022). Self-compassion – the skill of treating oneself kindly during moments of pain – is considered a crucial component of mindfulness interventions for chronic pain and has also been used to explore the mechanisms of mindfulness analgesia (Singer and Klimecki, 2014; Berry et al., 2020). It has been found that self-compassion training supports regulation of pain through the involvement of self-referential (ventral posterior cingulate cortex, vPCC), salience-processing (temporo-parietal junction, TPJ), and emotion regulatory (dlPFC) brain areas (Berry et al., 2020). However, the specific role of self-compassion as part of a mindfulness-training, and particularly in conjunction with the influence of other training factors, has not yet been determined in detail. However, it is also an important aspect that, for example, after self-compassion training, changes in the brain regions involved in self-compassion have also been found being altered in response to other self-referential cognitive processes, including interoceptive attention, cognitive reappraisal, and also in emotion regulation strategies (Berry et al., 2020). The analgesic effects of mindfulness may thus also relate to all these factors, which could significantly be involved in the pathway through which mindfulness impacts pain perception. Further studying these factors in the context of mindfulness and disentangling their respective processes more clearly, would help to unravel the mechanisms of analgesia based on mindfulness. This can aid in elucidating how the analgesic effect of one component of mindfulness or a specific psychological process induced or facilitated by mindfulness is achieved by eliciting specific neural changes. It may also help to identify potential mediators along the pathways of mindfulness-based analgesia. Therefore, future studies should consider equivalent subject conditions and experimental designs and perform studies in a step-by-step fashion comparing the effects and mechanisms of mindfulness training strategies in the context of different internal and external factors. Only this might help to clearly clarify which pathways and neural mechanisms are shared by mindfulness training strategies and which are specific to a particular factor and training component.

### 4.2. The brain-behavior correlations related to mindfulness analgesia may vary depending on different attention directions

As manipulation of mindfulness in previous studies on the mechanisms of mindfulness-based analgesia, can refer to either attending to pain or shifting the attention away from pain, another factor that may have contributed to the contradictory findings may be the attention direction. Some studies (Zeidan et al., 2011, 2015) examined the pain-relieving mechanism of mindfulness by instructing participants to turn their attention away from pain. They reported a reduction in both pain intensity and unpleasantness scores (associated with increased activation in the orbitofrontal, subgenual anterior cingulate, and anterior insular cortex). Other studies (Gard et al., 2012) have required participants to direct their attention toward the site of pain, and have not observed significant changes in pain intensity scores, but have reported significantly lower pain unpleasantness scores (associated with the increased activation in the pINS, and reduced activation in the lPFC) in the mindfulness group when compared with the control group.

When subjects completed a task in which their attention is directed away from pain (e.g., focus on breathing), they were actually completing an active distraction task (Perlman et al., 2010; Maser, 2012; Lomas et al., 2015). The mindful attitude (Wang et al., 2015) and the improved attention flexibility (Jha et al., 2007; Maser, 2012) as a result of mindfulness training enabled them to better keep their attention on the current task rather than being uncontrollably drawn to the sensation of pain. However, when subjects completed the mindfulness-based task of keeping attention on the site of pain, long-term mindfulness training enabled the mindfulness expert (no studies have yet shown that novice subjects can maintain their attention at the site of pain with a mindful attitude and report lower pain scores) to disassociate the pain perception process from cognitive-affective processing, achieving the goal of having pain processing based only on the current pain experience. Therefore, attention direction has a significant impact on the pathways and mechanisms underlying mindfulness-based analgesia.

### 4.3. Mindfulness may also indirectly influence pain perception by impacting emotional reactions and anticipation

Moreover, the model presented in this review provides a pathway of how mindfulness directly impacts pain perception, however, it is important to note that mindfulness may also indirectly influence pain perception by impact other factors. It has been found that mindfulness-related health benefits are associated with enhancements in cognitive control, emotion regulation, positive mood, and acceptance, each of which has been associated with pain modulation (Grossman et al., 2004). It has therefore been suggested that mindfulness attenuates pain through some of these processes. For example, it has been hypothesized that mindfulness training can attenuate pain by altering emotional responses to pain (Kabat-Zinn, 1982; Kabat-Zinn et al., 1985). One multivariate path analyses revealed that mindfulness reduced pain symptoms by reducing anxiety and emotional reactions to irritable bowel syndrome (IBS) symptoms (Garland et al., 2012).

In addition, a reduction in anticipation of processes and stimuli has been suggested as another possible mechanism for mindfulness-based analgesia (Brown and Jones, 2010). For example, when compared to controls, the mindfulness group exhibited smaller anticipation-evoked potentials in the right inferior parietal cortex and MCC, indicating less anticipation of the noxious stimuli, and lower activation in the MCC during anticipation (Brown and Jones, 2010). An fMRI-based study by Lutz et al. (2013) also found that mindfulness experts have lower levels of pain unpleasantness when compared with novices, which was associated with increased activation in the dorsal anterior insula and anterior middle cingulate gyrus (aMCC). This is inferred that mindfulness could reduce the anticipation of an aversive event and thus achieve an analgesic effect (Lutz et al., 2013).

Overall, the existence of these factors may reveal the indirect pathway of mindfulness-based analgesia, but it may also be an important reason for the inconsistent conclusions of previous studies on the mechanism of mindfulness-based analgesia.

## 5. Conclusions and future direction

In this publication, we presented a dynamic model that considers the potential moderating effect of mindfulness levels on analgesia. The model postulates that during the early stages of mindfulness practice, mindfulness analgesia may be achieved through an enhancement of cognitive regulation, while at later stages of mindfulness practice, any mindfulness-based analgesic effect may be achieved when the interference of cognitive-affective factors, often observed during the processing of pain, is reduced.

At the neural level, these dynamic changes may be characterized by short-term mindfulness training leading to a decrease in pain scores, accompanied by increased activation in cognitive-affective-related brain areas, such as the ACC and aINS. On the other hand, advanced training or individuals with higher trait mindfulness reporting lower pain scores may show increased activation in sensory processing-related brain areas, such as the pINS, and reduced activation in cognitive-affective-related areas, such as the lPFC.

Although clinical studies on the analgesic effects of mindfulness demonstrate significant improvement in chronic pain conditions, these studies also show some weaknesses which hinders robust conclusions. This is specifically attributed to the low quality of some studies including small sample sizes, lack of active control groups, insufficient statistical analysis, absence of follow-ups, which could all affect the effectiveness of mindfulness in alleviating chronic pain. Therefore, future research will need to design more rigorously and larger scaled RCTs to build a repository that can more decisively estimate the efficacy of mindfulness on chronic pain. Since RCTs are very complex and extensive, also a staggered approach might be meaningful. In this respect, it might also be of particular importance to consider follow-ups, for example 6 to 12 months after mindfulness training, to assess its long-term impact on chronic pain, and so further evaluate and disentangle underlying mechanisms. Additionally, compliance with mindfulness practice should be monitored during both the practice and follow-up processes, with multiple measurements of mindfulness levels and pain scores. This will help to address the unresolved question from previous studies about whether there is a minimum frequency or duration for mindfulness practice to be effective.

As for the pathways and neural mechanisms of mindfulness analgesia, previous research on the mechanisms of mindfulness analgesia has primarily focused on studies that assess these mechanisms before and after mindfulness training, or examine the differences between groups of mindfulness novices and expert practitioners. Consequently, these studies offer limited insight into the mechanisms during the mindfulness process itself and how these change due to internal and external factors and over time. Our theoretical model takes into account that mindfulness analgesia undergoes dynamic changes depending on the levels of mindfulness proficiency. This underscores the importance of exploring precise time points during mindfulness practice when potential shifts in brain and behavior might occur. Future studies could consider enrolling subjects who have no prior experience with mindfulness practice and setting up a series of assessments that encompass both behavioral and neuroscience data over a longer course of mindfulness training. For instance, conducting measurements on a weekly basis spanning from the initial assessment to the conclusion of the training period may provide valuable insights. In this respect it would be of particular importance to integrate various internal and external outcome measures that are subject to time changes themselves and could on the same time affect the time course of mindfulness training performance. Currently no research have conclusively specified the optimal duration for mindfulness training, which might therefore be a first angle to start the investigation of longitudinal processes and dynamics. It may not only help in unraveling the occurrence and progression of behavioral and neural changes induced by mindfulness, and so leading to a clearer comprehension of the pathways of mindfulness-based analgesia, but it may also hold the potential to steer the clinical application of mindfulness. For example, it may offer insights into the necessary intervention duration, frequency, and dosage for utilizing mindfulness as an intervention for chronic pain. With the integration of external and individual factors, one could make step into subgroup-specific indications and more precise treatments. This also relates to the fact that mindfulness-based analgesia may exert its effects through indirect pathways, engaging various components of mindfulness or psychological processes induced or facilitated by mindfulness, such as attention, self-compassion, anticipation, and emotional responses. Consequently, forthcoming studies investigating the junctures at which alterations occur in mindfulness analgesia should concurrently assess these components or processes alongside neural data and pain rating information. Additionally, researchers should delve into the correlation between these components or processes and the analgesic effect and mechanisms of mindfulness analgesia. This approach will not only help in understanding when these components or processes, undergo changes over time, but might also offer potential explanations for the dynamic shifts in mindfulness analgesia as it may change with advancing levels of mindfulness.

## Author contributions

CL: Conceptualization, Funding acquisition, Methodology, Writing – original draft. VM: Visualization, Writing – review & editing. FN: Conceptualization, Funding acquisition, Supervision, Writing – review & editing.
