# Supplementary material for: Dynamic processes of mindfulness-based alterations in pain perception
Source: Front Neurosci. 2023 Nov 9;17:1253559. doi: 10.3389/fnins.2023.1253559 (PMC10665508; doi:10.3389/fnins.2023.1253559)
Supplement: Supplementary file 1 [file Table_1.DOCX]

| Table 1 Characteristics of RCT trails of mindfulness and pain (Studies based on behavioural data only). | | | | | | | | |
| --- | --- | --- | --- | --- | --- | --- | --- | --- |
| Reference | No. of participants No. of groups | Mean age  ±SD  or age range | Inclusion criteria | Mindfulness group | Control group | Longest follow-up | Outcome measures | Effects on the outcome measures  /Results |
| Astin et al., 2003  The Journal of Rheumatology | 128 (2) | 47.7±10.6 | FM | MBSR & Qi Gong group:  2.5 h, every week, 8 weeks | Education support group:  Short lectures on a different topic, 2.5h, every week,8 weeks | 6 months | Fibromyalgia Impact Questionnaire, total myalgic Score, pain, and depression, and 6-minute walk | Both groups:  Fibromyalgia ↓  Myalgia ↓  Pain↓  Depression↓  No significant differences between groups |
| Bakhshani et al., 2015  Global Journal of Health Science | 40 (2) | MBSR 30.60±9.08  CG 31.50±9.57 | Chronic headache | MBSR group:  90 minutes, every week,8 weeks | Control group:  Usual pharmacotherapy (including specific and nonspecific drugs) | None | SF-36 questionnaire, and Headache log | Intervention group:  Quality of life↑  Pain ↓ |
| Banth & Ardebil, 2015  International Journal of Yoga | 88 (2) | Age 30–45 years | LBP, female | MBSR+ usual medical care group:  90 minutes, every week,8 weeks | Control group:  Medical care only. | 4 weeks | Mac Gil pain and standard brief quality of life scales | MBSR group:  Pain ↓  physical quality of life↑  mental quality of life↑ |
| Burnett et al., 2017  Pain Studies and Treatment | 24 (2) | Over 18 years of  age | Experimental acid pain | Mindfulness group: Practiced 10 minutes of mindfulness meditation following brief instructions provided by the researcher in between cold-pressor tasks. | Control group:  Seat quietly for 10 minutes in between cold presser tasks. | None | Anxiety towards pain, pain Threshold, pain Tolerance, pain Intensity,pain unpleasantness. | Mindfulness group:  Anxiety towards pain ratings ↓  pain threshold↑  pain tolerance↑  pain threshold times↑  tolerance times↑ |
| Cash et al., 2015  ann. behav. med. | treatment (n=51)  wait-list control participants (n=40) | Aged  18 years and older | Fibromyalgia sufferers | MBSR group:  2.5 hours, every week,8 weeks | Control group:  In a waiting list and were offered the MBSR program only after the  conclusion of the study. | 2 months | Perceived stress, pain, sleep quality, fatigue, symptom severity, and salivary cortisol | MBSR group:  perceived stress↓  sleep disturbance↓ symptom severity↓  Greater home practice at follow-up was associated with reduced symptom severity. |
| Cathcart et al., 2014  Behavioural and Cognitive Psychotherapy | MBT (n=23)  wait-list control participants (n=19) | MBT  45.78±13.1  CG  45.26±14.2 | CTH | MBT group:  3- week period involving twice-weekly group classes and daily practice. | Control group:  In a waiting list and were offered the MBSR program only after the  conclusion of the study. | None | Mindfulness ,  Headache,  Depression, Anxiety, Stress | MBT group:  headache frequency↓  mindfulness facet of observe↑ |
| Cherkin et al., 2016  Journal of the American Medical Association | MBSR  (n = 116),  CBT  (n = 113),  usual care (n = 113) | Aged 20 to 70 years | Chronic low back pain | 1. MBSR group:   (training in mindfulness meditation and yoga) delivered in 8 weekly 2-hour. | 1. CBT group:   (training to change pain-related thoughts and behaviours),8 weekly 2-hour;   1. Usual care group:   Included whatever care participants received. | 52 weeks. | Functional limitations and self-reported back pain at 26 weeks.  Outcomes were also assessed at 4, 8, and 52 weeks. | MBSR and CBT group:  functional limitations↓  back pain ↓ |
| Coelho et al., 2018  Breast Cancer Research and Treatment | 82 (2) | IG  44.7±15.9  CG  43.9±11.8 | Women undergoing percutaneous imaging-guided breast biopsy. | Mindfulness group:  Waited for the procedure in a quiet and silent room with  meditation videos, which associates relaxing music and nature  landscapes images. | The standard-care control group:  Remained in the waiting room watching a regular television program. | None | Depression, anxiety, stress, pain, systolic, diastolic blood pressure, initial and final temperate, heart rate, oxygen saturation, and salivary cortisol measured. | Mindfulness group:  perceived stress↓  blood pressure↓  heart rate↓  oxygen saturation↓ |
| Cooperman et al., 2021  Journal of Substance Abuse Treatment | 30 (2) | Age 18 or older | A) Receiving MMT for OUD,  B) Experiencing  at least mild, non-malignant, pain for at least three months. | MORE group:  Received their MMT, as usual, and attended eight, weekly,  two-hour MORE groups at their MMT clinics. | TAU group:  Received their MMT, as usual, and  group or individual counseling, as required by the clinic. TAU counseling consisted of relapse prevention,  cognitive-behavioral therapy, and supportive treatment. | 16 weeks. | Feasibility and engagement, drug use, craving, depression and anxiety, pain, health, and well-being. | MORE group:  baseline adjusted days of illicit drug↓  craving↓  pain↓  physical and emotional limitations↓  depression↓  anxiety↓  well-being↑  vitality↑  social functioning↑ |
| la Cour & Petersen, 2015  Pain Medicine | 109 (2) | MBSR  46.52±2.42  wait list  48.84±12.20 | Nonspecific chronic pain | MBSR group:  Participants were seen at weekly group meetings: Eight  meetings were 3-hour sessions, and one meeting was 4.5 hours. A follow-up session was conducted 2 months after  the last session. | Wait list group: Received treatment at the pain center as usual. This included going to scheduled meetings with physicians, nurses, psychologists, or social workers. The wait list period lasted between 2 and 2.5 months. After this period, the patients were assigned to the MBSR program, and program and measurements were performed exactly as in the treatment group. | 6 months | Pain, physical function, mental function, pain acceptance, and health-related quality of life.  The SF36 vitality scale was chosen as the primary outcome measure.  Data were compared at three time points: at baseline, after completion of the course/waiting period, and at the 6-month follow-up. | MBSR group:  anxiety↓  Depression↓  mental quality of life↑  feeling in control of the pain↑  pain acceptance↑ |
| Davis & Zautra, 2013  ann. behav. med. | 79 (2) | Being over 18 years of age | FM | MSER group:  The program was comprised of 12 modules, each lasting approxiately 15 min and centering on a particular topic, 6 weeks. | The HT condition group:  The health participants were given access to 12 modules of health behavior information across the 6 weeks of the intervention in a format similar to that of the MSER condition. | None | Pain, coping efficacy, affect, and social relations. | MSER group: Self efficacy for coping with pain↑  positive engagement in relationships↑  positive affect↑  relationship stress↓ |
| Day et al., 2013  Clin J Pain | intent-to-treat sample MBCT  (n= 17), intent-to-treat sample DT  (n= 17),  on the completer sample  MBCT(n = 9),  DT(n= 15) | 19 years of age or older | Headache pain | MBCT group:  8 weekly sessions (2 h each) and completed the daily online meditation practice and a daily headache  diary throughout thetreatment period. | Control group:  Continued medical treatment as usual and completed a daily headache  diary throughout the DT period. | None | Headache and preassessment and post assessment data | MBCT group:  self efficacy↑  pain acceptance↑  pain interference↓  pain catastrophizing↓ |
| Dowd et al., 2015  Clin J Pain | 124 (2) | Being over 18 years of age | Self-reported chronic pain  unrelated to cancer and of at least 6 months duration | MIA group:  12 sessions of treatment, twice per week for 6 weeks. Individual sessions were approximately of 20 minutes’ duration and each session also included a recommended audio-recorded meditation component that participants were asked to access daily. | PE group:  12 sessions of treatment, twice per week for 6 weeks. Individual sessions were approximately of 20 minutes’ duration. | 6 months | Physical functioning and disability, psychological  distress, pain intensity, participant ratings of improvement  and satisfaction with treatment, catastrophic thinking,  adherence to the treatment region, subjective well-being, pain acceptance, self-reported  mindfulness, pain interference, psychological  distress, pain on average  and pain right now. | Both groups:  pain interference↓ pain acceptance↑  pain catastrophizing↓  average pain intensity↓ |
| Duncan et al., 2017  BMC Pregnancy and Childbirth | 30 (2) |  | Women with low-risk, healthy, singleton pregnancies in their third trimester who were planning a hospital birth. | MIL group:  2.5-day mindfulness-based childbirth preparation course offered as a weekend workshop, the MIL: working with pain in childbirth, based on MBCP education. | TAU control group:  Provided with a list of study-approved childbirth courses of comparable length and quality to the MIL intervention, but without any mindfulness meditation, mindful movement/yoga, or other core mind/body component (e.g., hypnosis). | None | Childbirth self-efficacy, maladaptive pain appraisal, perceived pain in labor, use of pain medication in labor, birth satisfaction, depression, mindfulness and mindful body awareness. | MIL group:  childbirth self-efficacy↑  mindful body awareness↑  post-course depression symptoms↓  rate of opioid analgesia use↓ |
| Esch et al., 2017  Frontiers in Human Neuroscience | 31 (2) | At  least 18 years old | Experimental acute pain | Participants were trained in a combined breathing/mindfulness meditation technique (intervention group N = 16) for five consecutive days – i.e., five daily group  sessions of 1.5 h each. | Control group:  Received no intervention. | None | Pain tolerance, attention performance, self-perceived mindfulness. | Both groups:  pain tolerance↑.  Paradoxically, increases in pain tolerance occurred in both groups, and correlated with reported mindfulness. Naloxone showed a trend to decrease pain tolerance in both groups. |
| Esmer et al., 2011  The Journal of the American Osteopathic Association | 25 (2) | IG  55.2±11.2  CG  54.9±9.5 | FBSS | MBSR:  For 8 weeks, patients engaged in classroom learning once per  week for 1.5 to 2.5 hours. | The control group continued with  their traditional care as prescribed by their medical care  providers. | 12 weeks | Pain, quality of  life, functionality, analgesic use, and sleep quality. | MBSR group:  pain acceptance↑ quality of life↑  functional limitation↓  pain level↓  frequency of use and potency of analgesics used for pain↓  sleep quality↑ |
| Fogarty et al., 2015  Ann Rheum | 51 (2) | MBSR  55±13  CG  52±12 | RA | MBSR group:  For 8 weeks, patients engaged in classroom learning once per  week for 1.5 to 2.5 hours. | Control group:  The control group agreed that they could participate free of charge  in MBSR after all data collection was completed. | 2, 4 and 6 months. | RA disease activity, duration of early morning stiffness and pain. | MBSR group:  morning stiffness↓  pain↓ |
| Forsyth & Hayes, 2014  Psychol Rec | 58 (3) | 29.31±11.21 | Cold presser (experimental acid pain) | 1. Acceptance of thoughts:   listened to recorded instructions and then completed a second administration of the cold pressor task.   1. Mindfulness of breathing groups:   Listening to recorded instructions and then completed a second administration of the cold pressor task. | 1. Spontaneous coping group: received the same   instructions prior to the first cold pressor trial asking them to  use any coping method they preferred (spontaneous coping). | None | Acceptance of thoughts, mindful awareness of breathing, and spontaneous coping on both pain tolerance and pain threshold. | The acceptance of thoughts and mindfulness of breathing condition groups:  pain tolerance↑ |
| Garland et al., 2017  Journal of the Society for Social Work and Research | 115 (2) | MORE  49.3 ± 13.7  SG  47.4 ± 13.6 | Participant endorsement of prescription opioid use for pain relief on a daily or near-daily basis for at least 90 days, and self-reported chronic pain conditions | MORE group:  Involved eight weekly group  Sessions.Group sessions were 2 hours long. | Control group:  A master’s degree-level clinical social worker facilitated an 8-week SG consisting of 2-hour sessions in which participants expressed emotions and discussed topics related to chronic pain and opioid use/ misuse. | 3 months | Opioid misuse | MORE group:  opioid misuse↓  No changes in 2,000 ms opioid AB were observed. Decreases in 200 ms opioid AB over the course of treatment predicted reduced opioid misuse by 3-month follow-up. |
| Garland, Bryan, et al., 2017  Drug and Alcohol Dependence | 55 (2) | 48.9±11.6 | Opioid-treated chronic pain | MORE group:  8 weeks, 8-session intervention,2 h long/session. | The active control group:  8 weekly, 2-h SG sessions, in which a Master’s-level clinical social worker facilitated emotional expression and discussion of topics pertinent to chronic pain and opioid use/misuse. | None | Pain intensity and affective state. | MORE group:  pain↓  positive affect↑ |
| Garland, Froeliger, et al., 2014  Psychopharmacology | MORE (n=20)  SG (n= 29) | 46.6±13.9 | Chronic  pain and prescription opioid misuse | MORE group:  8 weeks, 8-session intervention,2 h long/session. | Active control group:  A master’s degree-level clinical social worker facilitated an 8-week SG consisting of 2-hour sessions in which participants expressed emotions and discussed topics related to chronic pain and opioid use/ misuse. | None | HR and HRV responses, and craving ratings | MORE group:  opioid misuse↓  opioid craving↓  subjective opioid cue-reactivity↓  HR decelerations↑  HRV↑  Within the MORE group, HR deceleration to natural reward cues was correlated with increased subjective arousal to the cues, whereas HR deceleration to opioid cues was correlated with decreased subjective arousal. Effects of MORE on craving were mediated by enhanced RR. |
| Garland, Hanley, Kline, et al., 2019  Drug and Alcohol Dependence | 30 (2) | Age 18 or older | OUD,  admitted to MMT  within the past year | MORE group:  8 weeks, 8-session intervention,2 h long/session. | TAU group:  Consisted of MMT in addition to individual and group therapy provided by participating MMT treatment agencies. TAU therapeutic approaches included process-oriented, present-centered therapy and cognitive-behavioral coping skills training but did not include formal mindfulness-based intervention. | None | Across 8 weeks of treatment, participants completed up to 112 random EMA  measures of craving, pain, and affect, as well as event-contingent craving ratings. Multilevel models examined the effects of MORE on craving, pain, and affect, as well as the association between positive affect and craving. | MORE group:  craving↓  pain unpleasantness↓  stress↓  positive affect↑  self-control↑  Positive affect was associated with reduced craving, an association that was significantly stronger among participants in MORE than TAU. |
| Garland et al., 2022  JAMA Internal Medicine | 250 (2) | 51.8±11.9 | Adults with chronic pain receiving long-term opioid therapy  who were misusing opioid medications. | MORE group:  8 weeks, 8-session intervention,2 h long/session. | Control group:  The supportive psychotherapy intervention involved discussions about coping with pain, the adverse effects of opioids, and the use of opioids to alleviate negative emotions. To match the homework requirement in the MORE group, participants in the supportive psychotherapy group were asked to write on weekly session topics in a journal for 15 minutes per day and to log minutes spent writing in their journal each day via their smartphones. | 9 months | Primary outcomes :  (1) opioid misuse (2) pain severity and pain-related functional interference. Secondary outcomes:  opioid dose, emotional distress, and ecological momentary assessments of opioid craving. | MORE group:  opioid misuse↓  pain severity↓  pain-related functional interference↓ |
| Garland et al., 2019  Journal of Consulting and Clinical Psychology | 95 (2) | 56.8±11.7 | Opioid-treated chronic, non-cancer pain,  female | MORE group:  8 weeks, 8-session intervention,2 h long/session. | Active control group:  Consisted of 8 weekly, 2-h SG sessions, in which a Master’s-level clinical social worker facilitated emotional expression and discussion of topics pertinent to chronic pain and opioid use/misuse. | 3 months | positive psychological health, pain  severity and opioid misuse risk. | MORE group:  Pain severity↓  opioid misuse risk↓  positive psychological health↑  Increases in positive psychological health mediated the effect of MORE on pain severity by post-treatment, which in turn predicted risk by follow-up. |
| Garland, Manusov, et al., 2014  Journal of Consulting and Clinical Psychology | 115 (2) | 48±14 | Chronic pain | MORE group:  8 weeks, 8-session intervention,2 h long/session. | Active control group:  Consisted of 8 weekly, 2-h SG sessions, in which a Master’s-level clinical social worker facilitated emotional  expression and discussion of topics pertinent to chronic  pain and opioid use/misuse. | 3 months | Pain severity and interference, Opioid use disorder status, Desire for  opioids, stress, nonreactivity, reinterpretation of pain sensations, and reappraisal. | MORE group:  pain severity and interference↓  nonreactivity and reinterpretation of pain sensations↑ |
| Garland et al., 2014  Journal of Pain and Symptom Management | 115 (2) | 48±14 | Chronic non-cancer-related pain and had been prescribed and taken opioids for analgesia daily or nearly every day for at least the past 90 days. | MORE group:  8 weeks, 8-session intervention,2 h long/session. | Control group:  The active control condition in this study consisted of eight weekly two hour conventional SG sessions | 3 months | Domains of pain-related functional interference and treatment effects. | MORE group:  functional interference↓  post-treatment across  all domains (general activity, mood, walking ability, normal work,  relationships, sleep, and enjoyment of life)↑ |
| Gaylord et al., 2011  American Journal of Gastroenterology | 75 (2) | Age 18–75 years | IBS patients，female | Mindfulness group:  Eight weekly and one  half-day intensive sessions of mindfulness-based stress and pain management  Program. | Control group:  As in the mindfulness intervention, the SG included one half-day session during the second half of the 8-week intervention. The half-day session involved the preparation and sharing of an “IBS-friendly” meal by the group participants. | 3 months | Participants completed the IBS severity scale (primary outcome), IBS-quality of life, brief symptom inventory-18, visceral sensitivity index, treatment credibility scale, and five-facet mindfulness questionnaire before and after treatment and at 3-month follow-up. | Mindfulness group:  IBS symptom severity↓ |
| Grossman et al., 2017  Clin J Pain | 130 (3) | 18 to 70 years of age | FM | A) MBSR group:  2.5 hours , every week,8 weeks | B) RELAX group:  8-week group interventions with groups of 10 to 15 women that met once a week for 2.5 hours.  C) wait-list | 8 weeks | Ambulatory accelerometry and cardiorespiratory function were monitored over 24-h periods at 3 time points. | Comparison with controls confirmed  differences in cardiac autonomic tone and activity pattern among  patients. Most measures also showed effects of time of day and point of measurement. |
| Jay et al., 2015  Pain Physician | 112 (2) | PCMT  45.5 ± 9.0  REF  47.6 ± 8.2 | Chronic musculoskeletal pain | PCMT group:  Consisted of 4 major elements:  1) individualized  motor control training 2) individualized resistance training specific to the pain affected area  3) cognitive and behavioral modification education emphasizing individual specific concerns about pain and movement,  and 4) general mindfulness.  10 weeks at the  worksite | REF group:  Received a single email after  randomization with encouragement to participate in the company’s on-going health initiatives, e.g., weekly elastic band group training sessions (only available in some departments) and was encouraged to continue to take “active breaks” whenever needed. As this is part of the existing and currently on-going program at the company it can be considered “usual care.” 10 weeks at the worksite. | 10 weeks | Pain and stress. | PCMT group:  Pain↓  A significant treatment by time interaction in pain intensity was observed with a between-group difference at follow-up of -1.0.  Within the PCMT group, general linear models adjusted for age, baseline pain, and stress levels showed significant associations for the change in pain with the number of physical-cognitive training sessions per week and the number of mindfulness session. |
| Johns et al., 2016  Support Care Cancer | 71 (2) | Age 18 or older | Breast and colorectal cancer survivors with clinically significant CRF after completing chemotherapy and/or radiation therapy an average of 28 months prior to enrollment. | MBSR group:  2 hours , every week,8 weeks | PES group:  For cancer survivors, PES programs include group discussions of CRF and its impact on psychological and social functioning; sharing, listening to, and affirming patients’ CRF-related experiences; and offering evidenced-based tips and strategies for managing CRF.2 hours , every week,8 weeks. | 6  months | CRF  interference (primary), CRF severity and global improvement, vitality, depression, anxiety, sleep disturbance, and pain. Outcomes were assessed at baseline (T1), post-intervention (T2), and 6-month follow-up (T3). | MBSR group:  pain↓ |
| Kanter et al., 2016  Int Urogynecol J. | 20 (2) | Age 18 or older | Women with IC/BPS undergoing 1st-or 2nd line therapies. | MBSR group:  8 weeks. The standardized course included seven 2-hr courses at weekly intervals with an all-day retreat in the 5th week. | Control group:  Continuation of UC | None | Participants completed baseline and 8-week posttreatment questionnaires including the OSPI, VAS, Short Form Health Survey (SF-12), FSFI, and PSEQ. GRA was completed post-treatment. | MBSR group:  GRA↑  OSPI↓  problem scores↓  PSEQ scores↑  Eighty-six percent of MBSR participants felt more empowered to control symptoms, and all participants planned to continue MBSR. |
| Kearney et al., 2016  The American Journal of  Medicine | 55 (2) |  | Gulf War Illness | MBSR group:  8 weekly 2.5 hour sessions plus a  single 7-hour weekend session. | Control group:  Treatment as usual. | 6 months | Pain, fatigue, and cognitive failures,symptoms of post traumatic stress disorder and depression. | MBSRgroup:  pain↓  Fatigue↓  cognitive failures↓ |
| Kerns et al., 2014  Health Psychology | 128 (2) | TCBT  55.5 ±13.1  SCBT  55.0 ±10.0 | Chronic back pain | TCBT group:  10 weekly, individual, 60-min outpatient sessions. A 14-week window for completion of the 10 sessions allowed for flexibility in scheduling. | SCBT group:  10 weekly, individual, 60-min outpatient sessions. A 14-week window for completion of the 10 sessions allowed for flexibility in scheduling. | None | Ratings of pain coping skill practice and goal accomplishment during treatment, as well as measures of pain severity, disability. | Both groups:  Ratings of pain coping skill practice↑  goal accomplishment↑ pain severity↓ disability↓  No significant differences between treatment groups were noted on measures of treatment engagement or adherence. |
| Kingston et al., 2007  Journal of Psychosomatic Research | 42 (2) | 23 | Experimental acid pain | Mindfulness group:  Six (1 h) mindfulness sessions | Control group:  Two (1 h) Guided Visual  Imagery sessions. | None | Pre–post pain tolerance, mood, blood pressure, pulse, and mindfulness skills. | Mindfulness group:  pain tolerance↑  mindfulness skills↑  Both group:  diastolic blood pressure↓  There was a strong  trend indicating that mindfulness skills increased in the mindfulness condition, but this was not related to improved pain tolerance. |
| Lengacher et al., 2016  J Clin Oncol | 322 (2) | age 21 years or  older | BCSs | MBSR(BC)group :  2-hour sessions once per week for 6 weeks conducted by  a clinical psychologist trained in MBSR and were provided training  manuals and CDs to guide their practice. | Usual care group:  standard posttreatment clinic visits and were offered the MBSR(BC) program on study  completion. | 12 weeks | Psychological (depression, anxiety, stress, and fear of recurrence) and physical symptoms (fatigue and pain) and quality of life (as related to health) were assessed at baseline and at 6 and 12 weeks. | MBSR(BC)group :  psychological symptoms of anxiety,  fear of recurrence↓ overall fear of recurrence problems↓  physical symptoms of fatigue severity↓ fatigue interference↓  Results demonstrated extended improvement for the MBSR(BC) group compared with usual care in both psychological symptoms of anxiety, fear of recurrence overall, and fear of recurrence problems and physical symptoms of fatigue severity and fatigue interference. |
| Liu et al., 2013  Stress and Health | 60 (3) | 20.48±1.47 | Experimental acid pain, female | A) Mindfulness group:  The instructions for mindfulness was given by recorded voices.15 minutes. | B) Mistraction group:  The instructions for distraction was given by recorded voices.15 minutes.  C) Spontaneous group:  No instruction was given. Instead, we played light music for the participants. 15 minutes. | None | Pain tolerance and  immersion distress. | Mindfulness group:  pain tolerance↑  immersion distress↓  Distraction group:  pain tolerance↑ |
| Ljótsson et al., 2010  Behaviour Research and Therapy | 86 (2) | 34.6±9.4 | IBS | Treatment group:  Consisted of a 10-week CBT protocol previously developed as a group treatment for IBS. The total time spent by the student therapists per  participant over the 10 weeks of treatment ranged between 8 min  and 315 min, with a mean of 165 min. | Wait list group. | 3 months | IBS-symptom severity, IBS-related quality of life, GI-specific anxiety, depression and general functioning. Participants were assessed at pre-treatment, post-treatment and 3-month follow-up (treatment condition only). | Treatment group:  primary IBS-symptoms↓  IBS-related quality of life↑  GI-specific anxiety↓ depression↓  general functioning↑  Control group:  primary IBS-symptom↑ |
| Ljótsson et al., 2011  Am J Gastroenterol | 195 (2) | 38.9±11.1 | IBS | ICBT group:  10 weeks and included an online therapist contact.The ICBT  emphasized acceptance of symptoms through exposure to IBS symptoms and related negative feelings.  The ICBT also included mindfulness training. | ISM group:  10 weeks and included an online therapist contact. The ISM emphasized symptom control through relaxation  techniques, dietary adjustments, and problem-solving skills. | 6 months | Severity of IBS symptoms, credibility of the treatments and expectancy of improvement. | At post-treatment and 6-month follow-up, significant differences on the GSRS-IBS, favoring ICBT were found. The difference on GSRS-IBS scores was 4.8 at post-treatment and 5.9 at 6-month follow-up. |
| Mascaro et al., 2021  Pain | 74 (2) | Mindfulness meditation intervention group  51.76±14.1  wait-list  54.15±15.3 | Self-reported chronic, distressing  levels of pain. | Mindfulness group: Provided a 1-year subscription to Headspace, a  meditation app that has more than 30 million worldwide  downloads. They were provided written instruction and assistance with downloading the app and were given the following directives: Please try to do one session or at least 10 minutes of meditation every day for 6 weeks. | Wait-list control group:  Received information about the app at the end of the 6-week study period. | None | Self reported pain severity, pain catastrophizing, and social and physical functioning. | Mindfulness group:  social functioning↑ |
| Meize-Grochowski et al., 2015  Geriatr Nurs | 27 (2) | 50 years of age or older | Postherpetic neuralgia | Treatment group:  Given an individual, one-hour session with one of the investigators, a certified MBSR instructor with more than 15 years’ experience and practiced daily meditation for six weeks. | Control group:  The PI telephoned all  participants weekly and reminded them verbally or through a phone  message to mail in their daily diaries each week. | None | Daily pain, fatigue levels, and meditation practice. | Treatment group:  neuropathic, affective,  and total pain↓  Control group:  affective pain↑ |
| Miller-Matero et al., 2019  Pain Medicine | 60 (2) | IG  55.21±15.94  CG  59.13±15.78 | Experiencing any level of acute pain during admission | Mindfulness group:  Mindfulness strategy  Once,10 minutes. | Control group:  Education on the Gate Control Theory of Pain Once,10 minutes. | None | Pain severity and stress. | Both groups:  pain↓ |
| Morone et al., 2016  JAMA Internal Medicine | 140 (2) | 65 years or older | CLBP | MBSR group:  8-week. To encourage proficiency with the meditation method after completion of the intervention, monthly 60-minute booster sessions were held. | Control group:  To ensure that the control group  received an equal amount of attention and social support, they also received monthly booster classes, which met  monthly for 1 hour. | 6 months | Primary outcome: functional limitations owing to LBP, pain (current, mean, and most severe in the past week) . Secondary outcomes:  quality of life, pain self-efficacy, and mindfulness. | MBSR group:  disability↓  severe pain↓ |
| Morone et al., 2008  Pain | 37 (2) | Aged 65  years and older | CLBP | MBSR:  Participants were seen in a group format once a week for 90 minutes for eight weeks. | Wait-list control group. | 3 months | Pain intensity,  pain acceptance, physical function, and quality of life. | Intervention group:  pain acceptance↑  activities engagement↑  physical function↑ |
| Morone et al., 2009  Pain Med | 40 (2) | Age ≥65 years | Moderate low back pain or greater for  at least the previous 3 months | MBSR group:  Classes met weekly for 8 weeks, and sessions lasted 90  minutes. | Control group:  8-week health education program on a successful aging curriculum that  has been used in other trials. For each class 45–60 minutes were devoted to a lecture given  by a heath professional on the topic for the session and 30–45 minutes (total 90 minutes) spent  doing a class “brain” exercise and discussion. | 4 months | Disability, psychological function, and pain severity. The same measures were obtained for both groups at baseline, at the end of the program, and 4 months after program completion. | Both groups:  disability↓  Pain↓  psychological function↑  The differences between the two groups did not reach statistical significance. |
| Omidi & Fatemeh Zargar, 2014 Nurs Midwifery Stud. | 60 (2) | The MBSR group:  34.5±2.41  The TAU group:  32±3.2 | Diagnosed with tension-type headache according to the International Headache Classification Subcommittee | MBSR group:  Received eight weekly treatments. Any session lasted 120 minutes. The sessions were based on MBSR protocol. | TAU group:  Treated by antidepressant medication and clinical management. | 3 months | Headache and mindfulness. | MBSR group:  pain severity↓  The results revealed the significant effect of time and interaction between time and type of treatment on the changes of scores. |
| Parisi et al., 2022  Behaviour Research and Therapy | 50  support group 45 | MORE group: 55.94 ±11.51  Support group: 57.64 ±11.95 | They reported recurring pain stemming from chronic, noncancer pain conditions; were prescribed and taking opioids analgesics daily or nearly every day for at least the past 90 days. | MORE group:  8 weeks. One of the few integrated treatments for opioid misuse and chronic pain. | Supportive group:  8 weeks psychotherapy. | None | Acute symptoms of pain, craving, negative affect, decentring, and curiosity. | MORE group:  acute symptoms of craving↓  negative affect↓  pain↓  MORE sessions produce cumulative benefits for such symptoms in everyday life over the course of treatment. |
| Parra-Delgado & Latorre-Postigo, 2013 Cognitive Therapy and Research | 33 (2) | 52.67±10.08 | Being diagnosed with fibromyalgia syndrome in accordance with the  diagnostic criteria proposed by the American College of Rheumatology and committing to the daily practice of mindfulness | MBCT group:  3 months, comprising eight structured group sessions each lasting 2 h 30 min. | Control group:  Received no intervention. At the end of the present study, those participants of the control group who did not meet inclusion criteria were invited to complete MBCT. | 3 months | Fibromyalgia Impact Questionnaire,  Beck Depression Inventory  and Visual Analogue Scale | MBCT group:  the impact of fibromyalgia↓  depressive symptoms↓  A slight decrease was observed in intensity of pain in different body areas although there were no significant differences between the groups. |
| Plews-Ogan et al., 2005  Journal of General Internal Medicine | 30 (3) | Average age was 46.5 | Musculoskeletal pain for greater than 3 months | MBSR group:  The participants  met weekly for eight 2.5-hour sessions. Meditation and yoga  practice. | Massage group:  One-hour massage sessions were given once per week for  8 weeks by 3 licensed massage therapists.  Standard care group:  At the 2 practices was to be seen by a primary care physician at least every 3 months with medication  adjustments made as indicated. | 12 weeks | Pain, physical, physical andand mental health status. | MBSR group:  pain unpleasantness↓  Massage group:  pain unpleasantness↓  mental health status↑ |
| Prins et al., 2014 European Journal of Pain | 46 (2) | 20.10±2.27 | Experimental acid pain | Mindfulness group:  Rre-recorded instructions were provided through headphones during the experimental pain induction. | Control group:  Distraction induction. | None | Pain catastrophizing and mindfulness. | No difference in experienced pain between the mindfulness group and the distraction group. However, a significant moderation effect was found. When dispositional pain catastrophizing was high, pain was less pronounced in the mindfulness group than in the distraction group, whereas the opposite effect was found when the level of pain catastrophizing was low. |
| Rahmani & Talepasand, 2015  Medical Journal of the Islamic Republic of Iran | 24 (2) | Experimental group 43.25±3.07  Control  group 44.8±3.28 | Patients diagnosed with stages l, ll, lll of breast cancer based on the clinical findings, cytological studies and diagnosis of a physician; the fatigue severity score in the patient had to be higher than 4; duration of breast cancer had to be more than a month | MBSR group:  Treatment was done in 8 group sessions. Eight intervention sessions of this study were followed based on the mindfulness-based  stress reduction program and were  conducted once a week in a 2-hour session for participants of the experimental group. | Control group:  Did not receive any interventions. Due to ethical considerations, participants of the control group were given a CD of the yoga practices at the end of the research. | 2 months | Overall quality of life, role, cognitive, emotion, social functions and pain and fatigue symptoms | MBSR group:  overall quality of life↑  role↑  cognitive↑ emotion↑  social functions↑ pain↓  fatigue symptoms↓ |
| Roberts et al., 2021  Journal of Psychosomatic Research | 95 (2) | 56.8±11.7 | Opioid-treated chronic pain | MORE group. | Support Group. | 3 months | Interoceptive awareness,  self-regulation, reappraisal,  distress. | MORE group:  interoceptive awareness↑  self-regulation↑  reappraisal↑  distress↓  Decreases in distress through 3-month follow-up were mediated by increases in reappraisal. |
| Schmidt et al., 2011 PAIN | 177 (3) | 52.5± 9.6 | Felmale FM paitients, 18–70 years,,  command of the German language and motivation to participate | A) MBSR group:  8-week structured program with groups of up to 12 patients, taught by a single instructor. Participants took part in one 2.5-h session every week, and an additional 7-h all-day session on a weekend day. | B) Active control group:  8-week group of size and weekly format similar to that of the MBSR program taught by a single instructor. In addition, equivalent amounts of social support and weekly topical educational discussions were provided. Use of PMR, and fibromyalgia-specific gentle stretching exercises served as counterparts for mindfulness and yoga elements of the MBSR curriculum.  C) Wait-list control group. | 8 weeks | HRQoL. | MBSR group:  HRQoL ↑  There were no significant differences between groups on primary outcome, but patients overall improved in HRQoL at short-term follow-up. |
| Sharpe et al., 2013European Journal of Pain | 138 (4) | Threat MIND 20.97±5.68  Threat PMR 19.57±3.04  No Threat MIND 19.94±3.41  No Threat PMR 19.74±2.99 | Consent, screening questionnaire,  Experimental acid pain | 1. Mindfulness meditation/threat group. 2. Mindfulness meditation/no threat group. | 1. Rogressive muscle relaxation/threat group. 2. Progressive muscle relaxation/no threat group. | None | DASS,  FPQ-III,  Threat manipulation check,  Toronto Mindfulness Scale,  Wells' Likert Scale,  Pain. | Threat group:  worry↑  fear of harm↑  expectations of pain↑  coping efficacy↓  Interaction effects revealed that mindfulness was effective in increasing curiosity and reducing decentring under conditions of high threat but not low threat. Other interactions on cognitive variables confirmed that mindfulness and relaxation appeared to exert influences under different conditions . Despite these cognitive effects being discerned under different conditions, there were no differences between mindfulness and relaxation on pain, tolerance or threshold in either threat group |
| Shires et al., 2019 European Journal of Pain | 100 (3) | Distraction group  20.54±4.436  Mindfulness group 19.50±3.194  Control group  19.80±3.002 | Experimental acid pain | 1. The first group: Participants received MIET instructions before the cold presser test. 2. The second group received distraction instructions prior to the cold presser test. | 1. The third group:   Completed the cold presser test without any specific instruction given. | None | Pain threshold, pain tolerance, distress at tolerance. | Participants who were allocated to the MIET condition reported a significantly higher pain threshold and distress than the distraction group, although not significantly higher than the control group. Difficulty disengaging from pain‐related stimuli, as measured by the duration of the first fixation on sensory words, was found to moderate the relative efficacy of mindfulness versus distraction in terms of pain threshold and distress, but not tolerance. |
| Soo et al., 2016  Journal of the American College of Radiology | 121 (3) | Meditation group:  56.10±13.04  Music group:  52.93±11.08  Standard care group:  49.85±12.78 | Female; CNBB. | 1. Meditation group:   Throughout the procedures, LKM patients listened to an audio-recorded adaptation of a commercially available LKM developed for this study, designed to help patients relax during CNBB, focusing on feelings of kindness and compassion toward themselves and others. | B) Music group:  Patients listened to their choice of instrumental jazz, classical piano, harp and flute, nature sounds, and world music.  C) Standard-care control group: Patients received supportive dialogue during CNBB, consisting of conversation initiated by the radiologist or technologist. “Where do you live?” was a common initial question; dialogue was not quantified or prescribed outside of normal practice. | None | State-trait anxiety, pain, functional assessment of chronic illness, fatigue, quality of physiciane patient Interaction, participant engagement and music interventions. | Meditation group:  anxiety↓  fatigue↓  pain↓  music group:  anxiety↓  fatigue↓  standard-care control group:  fatigue↑ |
| Swain & Trevena, 2014 New Zealand Journal of Psychology | 240 (2) | 21±2.98 | Experimental acid pain. | Experimental group:  Contact with therapist. | Control group:  DVD of therapist . | None | Pain tolerance | Both groups:  pain tolerance↑  Very brief interventions of both hypnosis and mindfulness were effective for acute pain management. |
| Teixeira, 2010  Holistic Nursing Practice | 22 (2) | Between the ages of 50 and 92 years | Symptoms of  PDPN | Treatment group:  Received instruction in mindfulness meditation and was instructed to listen to a guided compact disc 5 days per week over a 4-week period. | Control group:  Received nutritional information and was asked to maintain a food diary for 4 weeks. | None | QOL, pain, and sleep. | Meditation group:  discomfort with pain↓  pain intensity↑ |
| Wachholtz & Pargament, 2005  Journal of Behavioral Medicine | 84 (3) | 19.1±1.03 | Experimental acid pain | Secular Meditation group:  20 min per day for 2 weeks.  Spiritual Meditation  20 min per day for 2 weeks. | Relaxation group:  20 min per day for 2 weeks.  All three groups were given identical instructions about how to relax physically  and create a physically relaxing atmosphere. | None | The length of time that individuals kept their hand in the water bath. Pain, anxiety, mood, and the spiritual health were assessed following the two-week intervention. | Meditation group:  anxiety↓  positive mood↑  spiritual health↑  spiritual  experiences↑  Significant interactions occurred (time × group). Meditation group also tolerated pain almost twice as long as the other two groups |
| Wang et al., 2019  Mindfulness | 119 (4) | 21.08± 2.05 | Experimental acid pain | A) Pain-acceptance group:  Instructed to  accept their thoughts and feelings without being controlled by  them.  B) Pain-attention group:  Instructed to pay close attention to the situation. During the brief training, they were instructed to close their eyes and pay attention to their breath, continuously attending to each  breathing cycle, as well as the pause between breathing  cycles.  C) Combined acceptance and attention group: Instructed to use both the acceptance and attention strategies to cope with the pain. The order in which these strategies were taught was counter-balanced. | D) Control group:  Given neutral reading materials in general science. After the reading period, the participants were provided with general instructions for the cold  pressor task. | None | Pain tolerance, pain intensity, distress, threshold, and endurance time. | Acceptance strategy group:  pain endurance↑  tolerance↑  Combined acceptance and attention group:  pain endurance↑  tolerance↑  The acceptance group had longer pain endurance and tolerance times than the attention and control groups. |
| Wells et al., 2014  Headache | 19 (2) | IG  45.9±17  CG  45.2±12 | Diagnosis of migraine  with or without aura | MBSR group:  class met for 8-weekly 2-hour sessions, plus one “mindfulness retreat day” (6 hours) led by a trained instructor. | Usual care group. | 28 days | Migraine frequency, headache severity, duration, self-efficacy, perceived stress, migraine-related disability/impact, anxiety, depression, mindfulness, and quality of life. | MBSR group:  migraine disability↓  headache impact↓  mindfulness↑ |
| Westenberg et al., 2018  Clin Orthop Relat Res | 159 (2) | IG  54±15  CG  55±15 | Chronic pain | Mindfulness group:  A single-center, single-blind randomized controlled trial of the mindfulness-based video exercise. | Attention placebo  control group:  An educational pamphlet about pain and stress presented to patients to read over 60 seconds. | None | Pain intensity, emotion,  trait anxiety, feasibility, acceptability. | Mindfulness group:  pain intensity↓  state anxiety ↓  anxiety symptoms↓ depression↓  anger↓ |
| S. Y. Wong, 2009  Hong Kong Med J | 100 (2) | Aged 18 to 65 years | Chronic pain | MBSR group:  8 weeks. | Control group:  Education program,  8 weeks. | 3 and 6 months | Primary outcome measures: pain intensity. Secondary outcome measures: mood status and symptoms assessed, health-related quality of life. | Both groups:  pain intensity↓  physical and  mental health ↑ |
| Wong et al., 2011  Clin J Pain | 39 (2) | Aged 24 to 64 years | Chronic pain | MBSR group:  8 weekly group sessions, each of 2.5 hours, with a 7-hour “retreat” session. | MPI group:  8 weekly, 2.5-hour group sessions,which took the form of instructional lectures on basic understanding of chronic pain, factors that increase or decrease chronic pain, and effective ways for participants to signal their chronic pain to others. | 6 months | Pain, mood symptoms, and health-related quality of life. | Both groups:  pain intensity↓  pain-related distress↓ |
| Wren et al., 2019  Supportive Care in Cancer | 60 (3) | 21 years old or older | Core needle breast biopsy, received an abnormal biopsy result,  and underwent breast surgery,  female | A) Lovingkindness  Meditation group:  Participants were  asked to practice for up to 20 min daily, 2 weeks. | B) Music group:  participants were encouraged to listen to the music for up to 20 min at least once per day, 2 weeks.  C) Usual care group:  participants received supportive dialogue from the biopsy team during biopsy, the usual care in clinic, 2 weeks. | None | Anxiety, pain, fatigue, physiologic reactivity, and self-compassion occurred prior to patients’ biopsy, following biopsy, 1 week after receipt of biopsy results, and 1 week following breast surgery. | Loving kindness meditation group:  pain↓  self-compassion↑  heart rate↓  Music group:  Pain↓ |
| Zautra et al., 2008  Journal of Consulting and Clinical Psychology | 144 (3) | M  46.17±12.70  P  51.00±10.74  E  51.43±13.89 | RA | A) Mindfulness meditation and emotion regulation therapy (M):  8-week treatment period in weekly 2-hr sessions. | B) Cognitive behavioral therapy for pain (P) :  8-week treatment period in weekly 2-hr sessions.  C) Education-only group (E): 8-week treatment period in weekly 2-hr sessions. | 6 months | Pain and mitogen-stimulated IL-6, a proinflammatory cytokine. | P group:  pain control↑  IL-6↓  coping efficacy↑  M group:  coping efficacy↑  The relative value of the treatments varied as a function of depression history. |
| Zeidan et al., 2016  The Journal of Neuroscience | 95 (2) | 27±7 | Experimental acid pain | Mindfulness group:  4 separate days (20 m/d) of mindfulness-based mental training. | Control group:  Listening to an audio  recording of The Natural History of Selborne across 4 d (20 m/d). | None | Pain intensity and unpleasantness ratings. | Mindfulness group:  pain intensity↓  unpleasantness ↓ |
| Zgierska et al., 2016  Pain Medicine | 35 (2) | 51.8± 9.7 | Opioid-treated  CLBP | Meditation-CBT Group:  The intervention comprised eight weekly group sessions (meditation and CLBP-specific CBT  components) and 30 minutes/day, 6 days/week of at-home practice. | Control group:  Usual care for CLBP and opioid therapy management was  provided. | None | Pain severity and function/disability, pain acceptance, opioid dose, pain sensitivity to thermal stimuli, and serum pain-sensitive biomarkers. | Meditation-CBT  Group:  pain severity↓  pain sensitivity to thermal stimuli↓  Exploratory analyses suggested a relationship between the extent of meditation practice and the magnitude of intervention benefits. |

*Up or down arrows indicates to increase or decrease accordingly in the outcome measures.

***AB***-Attentional bias; ***ACT***-Acceptance and commitment therapy; ***AN****-*Attention Network Test; ***App****-*Mobile application; ***BC***-Breast cancer; ***BCS****s*-Breast cancer survivors; ***CBT***-Cognitive behavioural therapy; ***CD***-Compact disc; ***CES-D***-Center for Epidemiological Studies depression inventory; ***CG***-Control group; ***CLBP***-Chronic low back pain; ***CNBB***-(Stereotactic- and ultrasound-guided) core-needle breast biopsy; ***CRP***-C-reactive protein; ***CTH***-Chronic Tension-Type Headache; ***CRF***-Cancer-related fatigue; ***DASS***-Depression Anxiety Stress Scales; ***DAS28-CRP***-Disease Activity Score in 28 joints-C-reactive protein; ***DT***-Delayed treatment; ***EMA****-*Ecological momentary assessments; ***FBSS***-Failed back surgery syndrome; ***FIQ***-Fibromyalgia Impact Questionnaire; ***FM***-Fibromyalgia (syndrome); ***FMI***-Freiburg Mindfulness Inventory; ***FPQ-III***-Fear of Pain Questionnaire; ***FSFI***-Female Sexual Function Index; ***GCQ***-Giessen Complaint Questionnaire; ***GM****-*Guided mindfulness-based meditation; ***GRA***-Global Response Assessment; ***GSRS-IBS****-*Gastrointestinal Symptom Rating Scale modified for patients with IBS; ***HT***-Health Tips; ***HR***-Heart rate; ***HRQoL***-Health related quality of life; ***HRV***-Heart rate variability; ***IBS***-Irritable bowel syndrome; ***IC/BPS****-*Interstitial Cystitis/Bladder Pain Syndrome; ***ICBT****-*Internet-based cognitive behavioural therapy; ***IG***-Intervention group; ***IL-6***-Interleukin-6; ***ISM***-Internet stress management ; ***LBP***-Low back pain; ***LKM***-Loving-kindness meditation; ***LTOT****-*Long-term opioid therapy; ***MAAS***-Mindful Attention Awareness Scale; ***MAIA***-Multidimensional Assessment of Interoceptive Awareness; ***MANOVA***-Multivariate Analysis of Variance; ***MBCT****-*Mindfulness-based cognitive therapy; ***MBSR***-Mindfulness-Based Stress reduction; ***MBSR(BC)***-Mindfulness-Based Stress Reduction for Breast Cancer; ***MBT***-Mindfulness-based therapy; ***MCID***-Minimal clinically important difference; ***MG****-*Mindfulness group; ***MIA***-Mindfulness in action; *MIET*-Mindfulness‐based interoceptive exposure task; ***MIL****-*Mind in Labor; ***MIND****-*Mindfulness; ***MM***-Mindfulness mediation; ***MMT***-Methadone maintenance treatment; ***MOUD****-*Medication for opioid use disorder; ***MORE****-*Mindfulness-Oriented Recovery Enhancement; ***MPI***-Multidisciplinary pain intervention; ***MSER***-Mindful socioemotional regulation; ***NIH****-*Toolbox for measuring self-reported pain; ***NRS****-*Numeric rating scales; ***OUD***-Opioid use disorder; ***OSPI***-O’Leary-Sant Symptom Problem Index; ***Pain-EP***-Pain Evoked potential; ***PCMT***-Physical, cognitive, and mindfulness group-based training; ***PDPN****-*Painful diabetic peripheral neuropathy; ***PE***-Psychoeducation; ***PES***-Psychoeducation/support groups; ***PLC****-*The Quality of Life Profile for the Chronically Ill; ***PI***-Principal investigator; ***PMR***-Progressive Muscle Relaxation; ***PSEQ***-Pain Self-Efficacy Questionnaire; ***PSQI***-Pittsburgh Sleep Quality Index; ***PPS***-Pain Perception Scale; ***UC***-Usual care; ***QLQ-BR23****-*Questionnaire to Measure Special “Life Quality” of Patients with Breast Cancer; ***QLQ- C30****-*Questionnaire Measuring the Global “Life Quality” in Cancer Patients; ***QOL***-Quality of life; ***RA***-Rheumatoid arthritis; ***REF****-*Reference group; ***RELAX***-Relaxation intervention; ***RR***-Reward responsiveness; ***SBB***-Stereotactic breast biopsy; ***SC***-Standard care; ***SD***-Standard deviation; ***SF-12***-Short Form Health Survey; ***SF36***-Standardized, well-validated, multidimensional questionnaire measures health, level of function, and well-being for eight dimensions; ***SG***-Support group; ***SCBT***-Standard cognitive– behavioral therapy; ***STAI*** ***or STAI-State***-State-Trait Anxiety Inventory; ***TCBT***-Tailored cognitive– behavioral therapy; ***VAS****-*Visual analogue scale.

Astin, J. A., Berman, B. M., Bausell, B., Lee, ; W-L, Hochberg, ; M, & Forys, K. L. (2003). The Efficacy of Mindfulness Meditation Plus Qigong Movement Therapy in the Treatment of Fibromyalgia: A Randomized Controlled Trial. *The Journal of Rheumatology*, *30*(10), 2257–2262. www.jrheum.org

Bakhshani, N.-M., Amirani, A., Amirifard, H., & Shahrakipoor, M. (2015). The Effectiveness of Mindfulness-Based Stress Reduction on Perceived Pain Intensity and Quality of Life in Patients With Chronic Headache. *Global Journal of Health Science*, *8*(4), 142–151. https://doi.org/10.5539/gjhs.v8n4p142

Banth, S., & Ardebil, M. D. (2015). Effectiveness of mindfulness meditation on pain and quality of life of patients with chronic low back pain. *International Journal of Yoga*, *8*(2), 128. https://doi.org/10.4103/0973-6131.158476

Burnett, D., Phillips, G., & Tashani, O. A. (2017). The Effect of Brief Mindfulness Meditation on Cold-Pressor Induced Pain Responses in Healthy Adults. *Pain Studies and Treatment*, *05*(02), 11–19. https://doi.org/10.4236/pst.2017.52002

Cash, E., Salmon, P., Weissbecker, I., Rebholz, W. N., Bayley-Veloso, R., Zimmaro, L. A., Floyd, A., Dedert, E., & Sephton, S. E. (2015). Mindfulness Meditation Alleviates Fibromyalgia Symptoms in Women: Results of a Randomized Clinical Trial. *Annals of Behavioral Medicine*, *49*(3), 319–330. https://doi.org/10.1007/s12160-014-9665-0

Cathcart, S., Galatis, N., Immink, M., Proeve, M., & Petkov, J. (2014). Brief mindfulness-based therapy for chronic tension-type headache: A randomized controlled pilot study. *Behavioural and Cognitive Psychotherapy*, *42*(1), 1–15. https://doi.org/10.1017/S1352465813000234

Cherkin, D. C., Sherman, K. J., Balderson, B. H., Cook, A. J., Anderson, M. L., Hawkes, R. J., Hansen, K. E., & Turner, J. A. (2016). Effect of mindfulness-based stress reduction vs cognitive behavioral therapy or usual care on back pain and functional limitations in adults with chronic low back pain: A randomized clinical trial. *JAMA - Journal of the American Medical Association*, *315*(12), 1240–1249. https://doi.org/10.1001/jama.2016.2323

Coelho, B. A., Paiva, S. de P. C., & da Silva Filho, A. L. (2018). Extremely brief mindfulness interventions for women undergoing breast biopsies: a randomized controlled trial. *Breast Cancer Research and Treatment*, *171*(3), 685–692. https://doi.org/10.1007/s10549-018-4869-9

Cooperman, N. A., Hanley, A. W., Kline, A., & Garland, E. L. (2021). A pilot randomized clinical trial of mindfulness-oriented recovery enhancement as an adjunct to methadone treatment for people with opioid use disorder and chronic pain: Impact on illicit drug use, health, and well-being. *Journal of Substance Abuse Treatment*, *127*, 1–8. https://doi.org/10.1016/j.jsat.2021.108468

Davis, M. C., & Zautra, A. J. (2013). An online mindfulness intervention targeting socioemotional regulation in fibromyalgia: Results of a randomized controlled trial. *Annals of Behavioral Medicine*, *46*(3), 273–284. https://doi.org/10.1007/s12160-013-9513-7

Day, M. A., Thorn, B. E., Charles Ward, L., Rubin, N., Hickman, S. D., Scogin, F., & Kilgo, G. R. (2013). Mindfulness-based Cognitive Therapy for the Treatment of Headache Pain A Pilot Study. *The Clinical Journal of Pain*, *30*(2), 152–161. https://doi.org/doi: 10.1097/AJP.0b013e318287a1dc

Dowd, H., Hogan, M. J., McGuire, B. E., Davis, M. C., Sarma, K. M., Fish, R. A., & Zautra, A. J. (2015). Comparison of an online mindfulness-based cognitive therapy intervention with online pain management psychoeducation: A randomized controlled study. *Clinical Journal of Pain*, *31*(6), 517–527. https://doi.org/10.1097/AJP.0000000000000201

Duncan, L. G., Cohn, M. A., Chao, M. T., Cook, J. G., Riccobono, J., & Bardacke, N. (2017). Benefits of preparing for childbirth with mindfulness training: A randomized controlled trial with active comparison. *BMC Pregnancy and Childbirth*, *17*(1), 1–11. https://doi.org/10.1186/s12884-017-1319-3

Esch, T., Winkler, J., Auwärter, V., Gnann, H., Huber, R., & Schmidt, S. (2017). Neurobiological aspects of mindfulness in pain autoregulation: Unexpected results from a randomized-controlled trial and possible implications for meditation research. *Frontiers in Human Neuroscience*, *10*. https://doi.org/10.3389/fnhum.2016.00674

Esmer, G., Blum, J., Rulf, J., & Pier, J. (2011). Mindfulness-Based Stress Reduction for Failed Back Surgery Syndrome: A Randomized Controlled Trial. *The Journal of the American Osteopathic Association*, *110*(11), 646–652. https://doi.org/https://doi.org/10.7556/jaoa.2010.110.11.646

Fogarty, F. A., Booth, R. J., Gamble, G. D., Dalbeth, N., & Consedine, N. S. (2015). The effect of mindfulness-based stress reduction on disease activity in people with rheumatoid arthritis: A randomised controlled trial. In *Annals of the Rheumatic Diseases* (Vol. 74, Issue 2, pp. 472–474). BMJ Publishing Group. https://doi.org/10.1136/annrheumdis-2014-205946

Forsyth, L., & Hayes, L. L. (2014). The Effects of Acceptance of Thoughts, Mindful Awareness of Breathing, and Spontaneous Coping on an Experimentally Induced Pain Task. *Psychological Record*, *64*(3), 447–455. https://doi.org/10.1007/s40732-014-0010-6

Garland, E. L., Baker, A. K., & Howard, M. O. (2017). Mindfulness-oriented recovery enhancement reduces opioid attentional bias among prescription opioid-treated chronic pain patients. *Journal of the Society for Social Work and Research*, *8*(4), 493–509. https://doi.org/10.1086/694324

Garland, E. L., Bryan, C. J., Finan, P. H., Thomas, E. A., Priddy, S. E., Riquino, M. R., & Howard, M. O. (2017). Pain, hedonic regulation, and opioid misuse: Modulation of momentary experience by Mindfulness-Oriented Recovery Enhancement in opioid-treated chronic pain patients. *Drug and Alcohol Dependence*, *173*, S65–S72. https://doi.org/10.1016/j.drugalcdep.2016.07.033

Garland, E. L., Froeliger, B., & Howard, M. O. (2014). Effects of Mindfulness-Oriented Recovery Enhancement on reward responsiveness and opioid cue-reactivity. *Psychopharmacology*, *231*(16), 3229–3238. https://doi.org/10.1007/s00213-014-3504-7

Garland, E. L., Hanley, A. W., Kline, A., & Cooperman, N. A. (2019). Mindfulness-Oriented Recovery Enhancement reduces opioid craving among individuals with opioid use disorder and chronic pain in medication assisted treatment: Ecological momentary assessments from a stage 1 randomized controlled trial. *Drug and Alcohol Dependence*, *203*, 61–65. https://doi.org/10.1016/j.drugalcdep.2019.07.007

Garland, E. L., Hanley, A. W., Nakamura, Y., Barrett, J. W., Baker, A. K., Reese, S. E., Riquino, M. R., Froeliger, B., & Donaldson, G. W. (2022). Mindfulness-Oriented Recovery Enhancement vs Supportive Group Therapy for Co-occurring Opioid Misuse and Chronic Pain in Primary Care A Randomized Clinical Trial. *JAMA Internal Medicine*, *182*(4), 407–417. https://doi.org/10.1001/jamainternmed.2022.0033

Garland, E. L., Hanley, A. W., Riquino, M. R., Reese, S. E., Baker, A. K., Salas, K., Yack, B. P., Bedford, C. E., Bryan, M. A., Atchley, R., Nakamura, Y., Froeliger, B., & Howard, M. O. (2019). Mindfulness-oriented recovery enhancement reduces opioid misuse risk via analgesic and positive psychological mechanisms: A randomized controlled trial. *Journal of Consulting and Clinical Psychology*, *87*(10), 927–940. https://doi.org/10.1037/ccp0000390

Garland, E. L., Manusov, E. G., Froeliger, B., Kelly, A., Williams, J. M., & Howard, M. O. (2014). Mindfulness-oriented recovery enhancement for chronic pain and prescription opioid misuse: Results from an early-stage randomized controlled trial. *Journal of Consulting and Clinical Psychology*, *82*(3), 448–459. https://doi.org/10.1037/a0035798

Garland, E. L., Thomas, E., & Howard, M. O. (2014). Mindfulness-oriented recovery enhancement ameliorates the impact of pain on self-reported psychological and physical function among opioid-using chronic pain patients. *Journal of Pain and Symptom Management*, *48*(6), 1091–1099. https://doi.org/10.1016/j.jpainsymman.2014.03.006

Gaylord, S. A., Palsson, O. S., Garland, E. L., Faurot, K. R., Coble, R. S., Mann, J. D., Frey, W., Leniek, K., & Whitehead, W. E. (2011). Mindfulness training reduces the severity of irritable bowel syndrome in women: Results of a randomized controlled trial. *American Journal of Gastroenterology*, *106*(9), 1678–1688. https://doi.org/10.1038/ajg.2011.184

Grossman, P., Deuring, G., Walach, H., Schwarzer, B., & Schmidt, S. (2017). Mindfulness-Based Intervention Does Not Influence Cardiac Autonomic Control or the Pattern of Physical Activity in Fibromyalgia during Daily Life. *Clinical Journal of Pain*, *33*(5), 385–394. https://doi.org/10.1097/AJP.0000000000000420

Jay, K., Brandt Msc, M., Hansen, K., Sundstrup, E., Jakobsen, M. D., Sjøgaard, G., & Andersen, L. L. (2015). Effect of Individually Tailored Biopsychosocial Workplace Interventions on Chronic Musculoskeletal Pain and Stress Among Laboratory Technicians: Randomized Controlled Trial. *Pain Physician Journal*, *18*, 459–471. www.painphysicianjournal.com

Johns, S. A., Brown, L. F., Beck-Coon, K., Talib, T. L., Monahan, P. O., Giesler, R. B., Tong, Y., Wilhelm, L., Carpenter, J. S., von Ah, D., Wagner, C. D., de Groot, M., Schmidt, K., Monceski, D., Danh, M., Alyea, J. M., Miller, K. D., & Kroenke, K. (2016). Randomized controlled pilot trial of mindfulness-based stress reduction compared to psychoeducational support for persistently fatigued breast and colorectal cancer survivors. *Supportive Care in Cancer*, *24*(10), 4085–4096. https://doi.org/10.1007/s00520-016-3220-4

Kanter, G., Komesu, Y. M., Qaedan, F., Jeppson, P. C., Dunivan, G. C., Cichowski, S. B., & Rogers, R. G. (2016). Mindfulness-based stress reduction as a novel treatment for interstitial cystitis/bladder pain syndrome: a randomized controlled trial. *International Urogynecology Journal*, *27*(11), 1705–1711. https://doi.org/10.1007/s00192-016-3022-8

Kearney, D. J., Simpson, T. L., Malte, C. A., Felleman, B., Martinez, M. E., & Hunt, S. C. (2016). Mindfulness-based Stress Reduction in Addition to Usual Care Is Associated with Improvements in Pain, Fatigue, and Cognitive Failures Among Veterans with Gulf War Illness. *American Journal of Medicine*, *129*(2), 204–214. https://doi.org/10.1016/j.amjmed.2015.09.015

Kerns, R. D., Burns, J. W., Shulman, M., Jensen, M. P., Nielson, W. R., Czlapinski, R., Dallas, M. I., Chatkoff, D., Sellinger, J., Heapy, A., & Rosenberger, P. (2014). Can we improve cognitive-behavioral therapy for chronic back pain treatment engagement and adherence? A controlled trial of tailored versus standard therapy. *Health Psychology*, *33*(9), 938–947. https://doi.org/10.1037/a0034406

Kingston, J., Chadwick, P., Meron, D., & Skinner, T. C. (2007). A pilot randomized control trial investigating the effect of mindfulness practice on pain tolerance, psychological well-being, and physiological activity. *Journal of Psychosomatic Research*, *62*(3), 297–300. https://doi.org/10.1016/j.jpsychores.2006.10.007

la Cour, P., & Petersen, M. (2015). Effects of Mindfulness Meditation on Chronic Pain: A Randomized Controlled Trial. *Pain Medicine (United States)*, *16*, 641–652. https://doi.org/https://doi.org/10.1111/pme.12605

Lengacher, C. A., Reich, R. R., Paterson, C. L., Ramesar, S., Park, J. Y., Alinat, C., Johnson-Mallard, V., Moscoso, M., Budhrani-Shani, P., Miladinovic, B., Jacobsen, P. B., Cox, C. E., Goodman, M., & Kip, K. E. (2016). Examination of broad symptom improvement resulting from mindfulness-based stress reduction in breast cancer survivors: A randomized controlled trial. *Journal of Clinical Oncology*, *34*(24), 2827–2834. https://doi.org/10.1200/JCO.2015.65.7874

Liu, X., Wang, S., Chang, S., Chen, W., & Si, M. (2013). Effect of brief mindfulness intervention on tolerance and distress of pain induced by cold-pressor task. *Stress and Health*, *29*(3), 199–204. https://doi.org/10.1002/smi.2446

Ljótsson, B., Falk, L., Vesterlund, A. W., Hedman, E., Lindfors, P., Rück, C., Hursti, T., Andréewitch, S., Jansson, L., Lindefors, N., & Andersson, G. (2010). Internet-delivered exposure and mindfulness based therapy for irritable bowel syndrome - A randomized controlled trial. *Behaviour Research and Therapy*, *48*(6), 531–539. https://doi.org/10.1016/j.brat.2010.03.003

Ljótsson, B., Hedman, E., Andersson, E., Hesser, H., Lindfors, P., Hursti, T., Rydh, S., Rück, C., Lindefors, N., & Andersson, G. (2011). Internet-delivered exposure-based treatment vs. Stress management for irritable bowel syndrome: A randomized trial. *American Journal of Gastroenterology*, *106*(8), 1481–1491. https://doi.org/10.1038/ajg.2011.139

Mascaro, J. S., Singh, V., Wehrmeyer, K., Scott, B., Juan, J., McKenzie-Brown, A. M., Lane, O. P., & Haack, C. (2021). Randomized, wait-list–controlled pilot study of app-delivered mindfulness for patients reporting chronic pain. *PAIN Reports*, *6*(e924), 1–8. https://doi.org/10.1097/pr9.0000000000000924

Meize-Grochowski, R., Shuster, G., Boursaw, B., DuVal, M., Murray-Krezan, C., Schrader, R., Smith, B. W., Herman, C. J., & Prasad, A. (2015). Mindfulness meditation in older adults with postherpetic neuralgia: A randomized controlled pilot study. *Geriatric Nursing*, *36*(2), 154–160. https://doi.org/10.1016/j.gerinurse.2015.02.012

Miller-Matero, L. R., Coleman, J. P., Smith-Mason, C. E., Moore, D. A., Marszalek, D., & Ahmedani, B. K. (2019). A Brief Mindfulness Intervention for Medically Hospitalized Patients with Acute Pain: A Pilot Randomized Clinical Trial. *Pain Medicine (United States)*, *20*(11), 2149–2154. https://doi.org/10.1093/pm/pnz082

Morone, N. E., Greco, C. M., Moore, C. G., Rollman, B. L., Lane, B., Morrow, L. A., Glynn, N. W., & Weiner, D. K. (2016). A mind-body program for older adults with chronic low back pain a randomized clinical trial. *JAMA Internal Medicine*, *176*(3), 329–337. https://doi.org/10.1001/jamainternmed.2015.8033

Morone, N. E., Greco, C. M., & Weiner, D. K. (2008). Mindfulness meditation for the treatment of chronic low back pain in older adults: A randomized controlled pilot study. *Pain*, *134*(3), 310–319.

Morone, N. E., Rollman, B. L., Moore, C. G., Li, Q., & Weiner, D. K. (2009). A mind-body program for older adults with chronic low back pain: Results of a pilot study. *Pain Medicine*, *10*(8), 1395–1407. https://doi.org/10.1111/j.1526-4637.2009.00746.x

Omidi, A., & Fatemeh Zargar, ; (2014). Effect of Mindfulness-Based Stress Reduction on Pain Severity and Mindful Awareness in Patients With Tension Headache: A Randomized Controlled Clinical Trial. *Nurs Midwifery Stud*, *3*(3), 1–5. https://doi.org/10.17795/nmsjournal21136

Parisi, A., Hanley, A. W., & Garland, E. L. (2022). Mindfulness-Oriented Recovery Enhancement reduces opioid craving, pain, and negative affect among chronic pain patients on long-term opioid therapy: An analysis of within- and between-person state effects. *Behaviour Research and Therapy*, *152*. https://doi.org/10.1016/j.brat.2022.104066

Parra-Delgado, M., & Latorre-Postigo, J. M. (2013). Effectiveness of mindfulness-based cognitive therapy in the treatment of fibromyalgia: A randomised trial. *Cognitive Therapy and Research*, *37*(5), 1015–1026. https://doi.org/10.1007/s10608-013-9538-z

Plews-Ogan, M., Owens, J. E., Goodman, M., Wolfe, P., & Schorling, J. (2005). Brief Report: A pilot study evaluating mindfulness-based stress reduction and massage for the management of chronic pain. *Journal of General Internal Medicine*, *20*(12), 1136–1138. https://doi.org/10.1111/j.1525-1497.2005.0247.x

Prins, B., Decuypere, A., & van Damme, S. (2014). Effects of mindfulness and distraction on pain depend upon individual differences in pain catastrophizing: An experimental study. *European Journal of Pain (United Kingdom)*, *18*(9), 1307–1315. https://doi.org/10.1002/j.1532-2149.2014.491.x

Rahmani, S., & Talepasand, S. (2015). The effect of group mindfulness-based stress reduction program and conscious yoga on the fatigue severity and global and specific life quality in women with breast cancer. *Medical Journal of the Islamic Republic of Iran*, *29*. http://mjiri.iums.ac.ir

Roberts, R. L., Ledermann, K., & Garland, E. L. (2021). Mindfulness-oriented recovery enhancement improves negative emotion regulation among opioid-treated chronic pain patients by increasing interoceptive awareness. *Journal of Psychosomatic Research*, *152*. https://doi.org/10.1016/j.jpsychores.2021.110677

Schmidt, S., Grossman, P., Schwarzer, B., Jena, S., Naumann, J., & Walach, H. (2011). Treating fibromyalgia with mindfulness-based stress reduction: Results from a 3-armed randomized controlled trial. *Pain*, *152*(2), 361–369. https://doi.org/10.1016/j.pain.2010.10.043

Sharpe, L., Nicholson Perry, K., Rogers, P., Refshauge, K., & Nicholas, M. K. (2013). A comparison of the effect of mindfulness and relaxation on responses to acute experimental pain. *European Journal of Pain (United Kingdom)*, *17*(5), 742–752. https://doi.org/10.1002/j.1532-2149.2012.00241.x

Shires, A., Sharpe, L., & Newton John, T. R. O. (2019). The relative efficacy of mindfulness versus distraction: The moderating role of attentional bias. *European Journal of Pain (United Kingdom)*, *23*(4), 727–738. https://doi.org/10.1002/ejp.1340

Soo, M. S., Jarosz, J. A., Wren, A. A., Soo, A. E., Mowery, Y. M., Johnson, K. S., Yoon, S. C., Kim, C., Hwang, E. S., Keefe, F. J., & Shelby, R. A. (2016). Imaging-Guided Core-Needle Breast Biopsy: Impact of Meditation and Music Interventions on Patient Anxiety, Pain, and Fatigue. *Journal of the American College of Radiology*, *13*(5), 526–534. https://doi.org/10.1016/j.jacr.2015.12.004

Swain, N. R., & Trevena, J. (2014). A comparison of therapist-present or therapist-free delivery of brief mindfulness and hypnosis for acute experimental pain. *New Zealand Journal of Psychology*, *43*(3), 22–28.

Teixeira, E. (2010). The Effect of Mindfulness Meditation on Painful Diabetic Peripheral Neuropathy in Adults Older Than 50 Years. *Holistic Nursing Practice*, *24*(5), 277–283. https://doi.org/doi: 10.1097/HNP.0b013e3181f1add2

Wachholtz, A. B., & Pargament, K. I. (2005). Is spirituality a critical ingredient of meditation? Comparing the effects of spiritual meditation, secular meditation, and relaxation on spiritual, psychological, cardiac, and pain outcomes. *Journal of Behavioral Medicine*, *28*(4), 369–384. https://doi.org/10.1007/s10865-005-9008-5

Wang, Y., Qi, Z., Hofmann, S. G., Si, M., Liu, X., & Xu, W. (2019). Effect of Acceptance Versus Attention on Pain Tolerance: Dissecting Two Components of Mindfulness. *Mindfulness*, *10*(7), 1352–1359. https://doi.org/10.1007/s12671-019-1091-8

Wells, R. E., Burch, R., Paulsen, R. H., Wayne, P. M., Houle, T. T., & Loder, E. (2014). Meditation for migraines: A pilot randomized controlled trial. *Headache*, *54*(9), 1484–1495. https://doi.org/10.1111/head.12420

Westenberg, R. F., Zale, E. L., Heinhuis, T. J., Özkan, S., Nazzal, A., Lee, S. G., Chen, N. C., & Vranceanu, A. M. (2018). Does a brief mindfulness exercise improve outcomes in upper extremity patients? A randomized controlled trial. *Clinical Orthopaedics and Related Research*, *476*(4), 790–798. https://doi.org/10.1007/s11999.0000000000000086

Wong, S. Y. (2009). Effect of mindfulness-based stress reduction programme on pain and quality of life in chronic pain patients: a randomised controlled clinical trial. *Hong Kong Med J*, *15*(6), 13–14.

Wong, Y.-S. S., Chan, F. W.-K., Wong, R. L.-P., Chu, M.-C., Lam, Y.-Y. K., Mercer, S. W., & Ma, S. H. (2011). Comparing the Effectiveness of Mindfulness-based Stress Reduction and Multidisciplinary Intervention Programs for Chronic Pain A Randomized Comparative Trial. *Clin J Pain*, *27*(8), 724–734. www.clinicalpain.com

Wren, A. A., Shelby, R. A., Soo, M. S., Huysmans, Z., Jarosz, J. A., & Keefe, F. J. (2019). Preliminary efficacy of a lovingkindness meditation intervention for patients undergoing biopsy and breast cancer surgery: A randomized controlled pilot study. *Supportive Care in Cancer*, *27*(9), 3583–3592. https://doi.org/10.1007/s00520-019-4657-z

Zautra, A. J., Davis, M. C., Reich, J. W., Nicassio, P., Tennen, H., Finan, P., Kratz, A., Parrish, B., & Irwin, M. R. (2008). Comparison of Cognitive Behavioral and Mindfulness Meditation Interventions on Adaptation to Rheumatoid Arthritis for Patients With and Without History of Recurrent Depression. *Journal of Consulting and Clinical Psychology*, *76*(3), 408–421. https://doi.org/10.1037/0022-006X.76.3.408

Zeidan, F., Adler-Neal, A. L., Wells, R. E., Stagnaro, E., May, L. M., Eisenach, J. C., McHaffie, J. G., & Coghill, R. C. (2016). Mindfulness-meditation-based pain relief is not mediated by endogenous opioids. *Journal of Neuroscience*, *36*(11), 3391–3397. https://doi.org/10.1523/JNEUROSCI.4328-15.2016

Zgierska, A. E., Burzinski, C. A., Cox, J., Kloke, J., Stegner, A., Cook, D. B., Singles, J., Mirgain, S., Coe, C. L., & Bačkonja, M. (2016). Mindfulness meditation and cognitive behavioral therapy intervention reduces pain severity and sensitivity in opioid-treated chronic low back pain: Pilot findings from a randomized controlled trial. *Pain Medicine (United States)*, *17*(10), 1865–1881. <https://doi.org/10.1093/pm/pnw006>

| Table 2 Characteristics of RCT trails of mindfulness and pain (Neurological mechanism-based studies). | | | | | | | | | |
| --- | --- | --- | --- | --- | --- | --- | --- | --- | --- |
| Reference | No. of participants No. of groups | Mean age  ±SD  or age range | Inclusion criteria | Mindfulness group | Control group | Longest follow-up | Outcome measures | Brain regions showing pain-related effects | Effects on the outcome measures  /Results |
| Braden et al., 2016  Brain and Behavior | 23 (2) | MBSR  46.0±11.3  CG 43.0±2.5 | Chronic low back pain | MBSR group:  2 h, every week,4 weeks. | Control group:  The CG never met together in person for any training session, but rather participants were  given a reading material, “Relaxation Techniques for Health: An Introduction” | 1 year | Self-report ratings and task-based functional  MRI | Frontal lobe | Both groups showed significant improvement in total depression symptoms, but only the MBSR group significantly improved in back pain and somatic-affective depression symptoms.  The MBSR group also showed significant increases in regional frontal lobe hemodynamic activity associated with gaining awareness to changes in emotional states. |
| Brown & Jones, 2012  Clin J Pain | 28 (2) | MBPM  48±10  CG  45±12 | Any type of  musculoskeletal pain | MBPM group:  2.5 hours, every week,8 weeks . | Control group: Treatment-as-usual | None | Clinical pain, perceived control over pain, mental and physical health, and mindfulness. Neural activity was measured during the anticipation and experience of acute experimental pain, using electroencephalography with source reconstruction. | Left dorsolateral prefrontal cortex and right secondary somatosensory cortex | Improvements in perceived control of pain were found in the MBPM group relative to the control group in mental health.The MBPM and control groups showed opposite pre to-post session effects on the anticipatory-evoked and pain evoked ERP response, with increases in both components in the control group and decreases in both components in the intervention group. Improvements in mental health were significantly predicted by less of a decrease in activity in left dorsolateral prefrontal cortex and right secondary somatosensory cortex during early anticipation of pain. |
| Day et al., 2021  Pain Medicine | 57 (3) | Being over 18 years of age | CLBP | A) MM group:  Only mindfulness meditation practices were taught, consisted of 8 weekly 2-hour sessions.  B) Cognitive and behavioral techniques group:  Cognitive and behavioral techniquesas well as mindfulness-based strategies were taught within the integrated MBCT condition, consisted of 8 weekly 2-hour sessions. | C) Cognitive therapy group:  Training participants in CT techniques  Only,consisted of 8 weekly 2-hour sessions. | None | Characteristic pain intensity,  brain oscillation bandwidth power,  EEG data were examined for five regions of interest (ROIs),  the primary outcome was pain intensity. | Five ROIs  **frontal left** (AF3, F3, F5, FC5),  **frontal right** (AF4, F4, F6, FC6),  **central left** (C3, C5, CP3, CP5, P3, P5), **central right** (C4, C6, CP4, CP6, P4, P6), **central** (FCz, Cz, C1, C2) | A significant reduction in theta and alpha power in the left frontal ROI across all treatments was found, although change in theta and alpha power in this region was not differential associated with outcome across treatments. There were significant reductions in beta power in all five ROIs across all treatments. Beta power reduction in the central ROI showed a significant association with reduced pain intensity in MBCT only. Changes in other regions were not statistically significant. |
| Garland et al., 2015  JBehav Med | MORE (n = 11) ， support group control  condition (n = 18) | 47.1±SD | Chronic pain and prescription opioid misuse | MORE group:  8 weeks, 8-session MORE intervention,2 h long/session. | Active control group:  8 weekly, 2-h SG sessions, in which a Master’s-level clinical social worker facilitated emotional  expression and discussion of topics pertinent to chronic  pain and opioid use/misuse. | None | ERPs to images representing naturally  rewarding stimuli (e.g., beautiful landscapes, intimate  couples) and neutral images were measured before and  after 8 weeks of treatment. | ERP was recorded from Pz | Treatment with MORE was associated with significant increases in LPP response to natural reward stimuli relative to neutral stimuli which were correlated with enhanced positive affective cue responses and reductions in opioid craving from pre- to post-treatment. |
| Garland & Howard, 2018  Journal of the Society for Social Work and Research | 29 (2) | 47.1±15.2 | Opioid-treated chronic pain patients | The manualized MORE intervention involved eight weekly group  Sessions. Group sessions were 2 hours long. | Active control group:  A master’s degree-level clinical social worker facilitated an 8-week SG consisting of 2-hour sessions in which participants expressed emotions and discussed topics related to chronic pain and opioid use/ misuse. | 3 months | At pretreatment and posttreatment, participants completed an affective picture viewing task while their EEG was recorded, and they completed self-report measures of pain severity, pain interference, and pain coping. Pain severity and interference were also reported at a 3-month posttreatment follow-up; pain coping was not measured at follow-up. | ERP was recorded from Pz | Increases in ERP reward response were significantly associated with decreased pain severity from pretreatment to posttreatment and improvements in pain catastrophizing and diverting attention as a means of pain coping. Increased ERP reward response predicted decreased pain interference by 3-month follow-up |
| Mioduszewski et al.,  Journal of Cancer Survivorship | 23 (2) | 52.78±10.77 | Breast cancer survivors experiencing chronic neuropathic pain,  female | MBSR group:  Once every week for a duration of 8 weeks. Each  weekly session lasted 2.5 h; additionally, one full-day session  was offered during the 5th week of the program. | Control group:  Waitlist. | None | Participants were imaged with MRI prior to and post-MBSR training using diffusion tensor imaging. | Left subcortical regions including the uncinate fasciculus, amygdala, and hippocampus, as well as in the external capsule and in the left sagittal stratum. | Compared with controls, the MBSR group showed a significant increase in FA, particularly in the  left subcortical regions including the uncinate fasciculus, amygdala, and hippocampus, as well as in the external capsule and in the left sagittal stratum. The FA values also  negatively correlated with the pain severity and pain interference scores from the brief pain questionnaire. |
| Smith et al., 2021 Journal of Cancer Survivorship | 23 (2) | 52.7±10.77 | Women suffering from CNP.  MRI compatibility criteria, agreed to be scanned, were right handed, and did not have hand pain (as they were required to use their hands for another fMRI task) | MBSR group:  In person program, included 8 weeks of one 2.5-h session/week with one full day session near the end of the program. Each session included a variety of mindfulness activities, including meditation practices, breath awareness, body scans, yoga, and walking meditation, among others. | Control group:  Waitlist. | None | BPI,  FFMQ,  fMRI | Posterior cingulate and medial prefrontal regions | Focusing on the default mode network, the most significant results show greater posterior cingulate connectivity with medial prefrontal regions post-MBSR intervention. Moreover, this change in connectivity correlated with reduced pain severity for the MBSR group. |
| Zeidan et al., 2015  The Journal of Neuroscience | 75 (4) | Mindfulness  28.06±6.75  Placebo  27.42±5.22  Sham  25.95±4.8  Book-listening control intervention  27.84±6.92 | Experimental acid pain | A) Mindfulness group: Mindfulness meditation 4 day, four training sessions (20 m/d). | B) Placebo group.  C) Sham mindfulness group.  D) Book-listening group. | None | The FMI, perceived intervention effectiveness was assessed with a VAS, fMRI acquisition. | The orbitofrontal, subgenual anterior cingulate, and anterior insular cortex. | Mindfulness meditation reduced pain intensity and pain unpleasantness ratings more than placebo analgesia and sham mindfulness meditation. Mindfulness-meditation-related pain relief was associated with greater activation in brain regions associated with the cognitive modulation of pain, including the orbitofrontal, subgenual anterior cingulate, and anterior insular cortex. |

***BPI***-Brief Pain Inventory; ***CG***-Control group; ***CLBP***-Chronic low back pain; ***CNP***-chronic neuropathic pain; ***CT***-Cognitive therapy; ***EEG****-*Electroencephalography; ***ERP***-Event-related potentials; ***FA***-Fractional anisotropy; ***FFMQ***-Five-Facet Mindfulness Questionnaire; ***fMRI***-Functional magnetic resonance imaging; ***LPP***-Late positive potential; ***MBCT****-*Mindfulness-based cognitive therapy; ***MBPM****-*Mindfulness-based pain management programs; ***MBSR***-Mindfulness-Based Stress reduction; ***MM***-Mindfulness mediation; ***MORE****-*Mindfulness-Oriented Recovery Enhancement; ***MRI****-*Magnetic Resonance Imaging; ***ROI***-Region of interest; ***SG***-Support group; ***VAS****-*Visual analogue scale.

Braden, B. B., Pipe, T. B., Smith, R., Glaspy, T. K., Deatherage, B. R., & Baxter, L. C. (2016). Brain and behavior changes associated with an abbreviated 4-week mindfulness-based stress reduction course in back pain patients. *Brain and Behavior*, *6*(3), 1–13. https://doi.org/10.1002/brb3.443

Brown, C. A., & Jones, A. K. P. (2012). Psychobiological Correlates of Improved Mental Health in Patients With Musculoskeletal Pain After a Mindfulness-based Pain Management Program. *The Clinical Journal of Pain*, *29*(3), 233–244. https://doi.org/DOI: 10.1097/AJP.0b013e31824c5d9f

Day, M. A., Matthews, N., Mattingley, J. B., Ehde, D. M., Turner, A. P., Williams, R. M., & Jensen, M. P. (2021). Change in Brain Oscillations as a Mechanism of Mindfulness-Meditation, Cognitive Therapy, and Mindfulness-Based Cognitive Therapy for Chronic Low Back Pain. *Pain Medicine (United States)*, *22*(8), 1804–1813. https://doi.org/10.1093/pm/pnab049

Garland, E. L., Froeliger, B., & Howard, M. O. (2015). Neurophysiological evidence for remediation of reward processing deficits in chronic pain and opioid misuse following treatment with Mindfulness-Oriented Recovery Enhancement: exploratory ERP findings from a pilot RCT. *Journal of Behavioral Medicine*, *38*(2), 327–336. https://doi.org/10.1007/s10865-014-9607-0

Garland, E. L., & Howard, M. O. (2018). Enhancing natural reward responsiveness among opioid users predicts chronic pain relief: EEG analyses from a trial of mindfulness-oriented recovery enhancement. *Journal of the Society for Social Work and Research*, *9*(2), 285–303. https://doi.org/10.1086/697685

Mioduszewski, O., Hatchard, T., Fang, Z., Poulin, P., Khoo, E.-L., Romanow, H., Shergill, Y., Tennant, E., Schneider, M. A., Browne, N., & Smith, A. M. (2020). Breast cancer survivors living with chronic neuropathic pain show improved brain health following mindfulness-based stress reduction: a preliminary diffusion tensor imaging study. *Journal of Cancer Survivorship*, *14*, 915–922. https://doi.org/10.1007/s11764-020-00903-w/Published

Smith, A. M., Leeming, & A., Fang, & Z., Hatchard, & T., Mioduszewski, & O., Schneider, M. A., Ferdossifard, & A., Shergill, Y., Khoo, & E.-L., & Poulin, & P. (2021). Mindfulness-based stress reduction alters brain activity for breast cancer survivors with chronic neuropathic pain: preliminary evidence from resting-state fMRI. *Journal of Cander Survivorship*, *15*, 518–525. https://doi.org/10.1007/s11764-020-00945-0/Published

Zeidan, F., Emerson, N. M., Farris, S. R., Ray, J. N., Jung, Y., McHaffie, J. G., & Coghill, R. C. (2015). Mindfulness meditation-based pain relief employs different neural mechanisms than placebo and sham mindfulness meditation-induced analgesia. *Journal of Neuroscience*, *35*(46), 15307–15325. https://doi.org/10.1523/JNEUROSCI.2542-15.2015
